# Odoriferous Defensive Stink Gland Transcriptome to Identify Novel Genes Necessary for Quinone Synthesis in the Red Flour Beetle, *Tribolium castaneum*

**DOI:** 10.1371/journal.pgen.1003596

**Published:** 2013-07-11

**Authors:** Jianwei Li, Sabrina Lehmann, Bernhard Weißbecker, Irene Ojeda Naharros, Stefan Schütz, Gerrit Joop, Ernst A. Wimmer

**Affiliations:** 1 Department of Developmental Biology, Johann-Friedrich-Blumenbach-Institute of Zoology and Anthropology, GZMB, Ernst-Caspari-Haus, Georg-August-University Göttingen, Göttingen, Germany; 2 Department of Forest Zoology and Forest Conservation, Büsgen-Institute, Georg-August-University Göttingen, Göttingen, Germany; 3 Department of Evolutionary Ecology and Genetics, Zoological Institute, Christian-Albrechts-Universität zu Kiel, Kiel, Germany; Janelia Farm Research Campus, Howard Hughes Medical Institute, United States of America

## Abstract

Chemical defense is one of the most important traits, which endow insects the ability to conquer a most diverse set of ecological environments. Chemical secretions are used for defense against anything from vertebrate or invertebrate predators to prokaryotic or eukaryotic parasites or food competitors. Tenebrionid beetles are especially prolific in this category, producing several varieties of substituted benzoquinone compounds. In order to get a better understanding of the genetic and molecular basis of defensive secretions, we performed RNA sequencing in a newly emerging insect model, the red flour beetle *Tribolium castaneum* (Coleoptera: Tenebrionidae). To detect genes that are highly and specifically expressed in the odoriferous gland tissues that secret defensive chemical compounds, we compared them to a control tissue, the anterior abdomen. 511 genes were identified in different subtraction groups. Of these, 77 genes were functionally analyzed by RNA interference (RNAi) to recognize induced gland alterations morphologically or changes in gland volatiles by gas chromatography-mass spectrometry. 29 genes (38%) presented strong visible phenotypes, while 67 genes (87%) showed alterations of at least one gland content. Three of these genes showing quinone-less (ql) phenotypes – *Tcas-ql VTGl*; *Tcas-ql ARSB*; *Tcas-ql MRP* – were isolated, molecularly characterized, their expression identified in both types of the secretory glandular cells, and their function determined by quantification of all main components after RNAi. In addition, microbe inhibition assays revealed that a quinone-free status is unable to impede bacterial or fungal growth. Phylogenetic analyses of these three genes indicate that they have evolved independently and specifically for chemical defense in beetles.

## Author Summary

Insects use chemical defensive secretions to defend themselves from all the potential detrimental factors in their surroundings. Tenebrionid beetles produce as irritants and repellents several varieties of substituted benzoquinone compounds that are highly reactive and toxic. However, the molecular basis of the controlled synthesis and secretion remains to be identified. In this study, we employ a newly emerging insect model, the red flour beetle *Tribolium castaneum*, to get a better understanding of the genetic and molecular basis of quinone production with identification of the highly and specifically expressed genes in secretory glands. By performing RNA sequencing and transcriptomics of different gland tissues, we identified 511 gland-specific genes. Of these, 77 genes were functionally examined for gland morphological changes and alterations in gland volatile composition. We showed that 67 genes (87%) cause alterations of at least one gland volatile after RNA interference-based gene knock-down. Three novel genes with quinone-less phenotypes were characterized further to confirm their importance in defensive secretions and their independent evolutionary origin.

## Introduction

Insects are among the most diverse group of animals on the planet and amazingly include more than a million described species, which is more than half of all known living organisms [1], [2]. Moreover, they have conquered almost every environment on earth. From a series of distinctive attributes that orchestrate together to endow them with the ability to live in a wide range of ecological environments, chemical defense is one of the most important traits [3]. Many chemical secretions have repellent or irritant properties [4], [5]. Tenebrionid beetles are especially prolific by producing several various substituted benzoquinone compounds [5]–[9]. *Tribolium* beetles (Coleoptera: Tenebrionidae) have dragged attentions of researchers to their particular secretions, since it was noted that their flour medium turns pink over time due to the secretion of a gaseous substance from adults [10], which also deleteriously affects the viscous and elastic properties of dough made from such infested flour [11].

*Tribolium* beetles possess for the purpose of chemical defense two pairs of specialized secretory organs – one in the prothorax and one in the posterior abdomen – termed odoriferous or stink glands [12]. The glands located in the prothorax are called prothoracic, thoracic, or anterior glands, while the other pair in the abdomen is referred to as abdominal, posterior, or pygidial glands. The fine structure of these glands revealed two types of secretory units composed of two slightly different types of cells with particular vesicular organelles (cell type 1 and cell type 2), tubules, reservoir, ducts and muscles [12]–[14]. At least four members of the genus *Tribolium (T. anaphe, T. castaneum, T. confusum*, and *T. destructor)* use the glands to produce the quinone derivatives 2-methoxybenzoquinone, ethyl-1,4-benzoquinone (EBQ), and methyl-1,4-benzoquinone (MBQ) [9], [12], [14]–[18]. However, only the latter two substances were detected in *T. confusum* [4], [6]. Besides benzoquinone derivatives, hydrocarbons were also reported as major secretion components. *T. confusum* secrets 1-pentadecene [19], 1,6-pentadecadiene and smaller amounts of 1-hexadecene, 1,6-hexadecadiene, hexadecatriene, 1-heptadecene, 1,8-heptadecadiene and heptadecatriene [20], [21]. In *T. castaneum*, 1-pentadecene and 1,6-pentadecadiene were identified, with the former as the main component [9], [18], plus two still unidentified hydrocarbons were found [18].

The red flour beetle, *T. castaneum* has been developed into a highly sophisticated genetic model organism [22] with plenty of genetic and genomic tools: reverse genetics based on systemic RNA interference [23], [24], forward genetics based on insertional mutagenesis [25], [26], transgene-based mis-expression systems [27], [28], as well as a fully annotated genome sequence [29]. Moreover, several mutants with odoriferous gland phenotypes, such as *melanotic stink glands* (*msg*, with both pairs of glands melanized) [30], *tar* (only prothoracic glands are darkly pigmented), and *box* (*A^box^*, similar to *tar*, but only the abdominal glands are affected) [31] are known. For quinone biosynthesis, it has been reported in another quinone-producing tenebrionid beetle *Eleodes longicollis* that alkylated benzoquinones are formed by acetate condensation whereas the p-benzoquinones are generated from preformed aromatic rings of amino acids (tyrosine and phenylalanine) [5], [32], [33]. In the glandular secretory cells, p-quinones are in a form of phenolic ß-glucoside contained in the more apical part, which were then transferred to inner part of the gland and form active quinones by a series of enzymatic reactions [14]. The alkenes are biosynthesized from fatty acids by oxidative decarboxylation [21]. However, no data are available on the genes involved in these processes. In addition, arthropods have evolved mechanisms to reduce the autointoxicative effects of a variety of toxic compounds produced by themselves for defense purpose [5]. For example, a paradoxsomatid millipede, *Oxidus gracilis*, which produces toxic phenol from tyrosine, possesses the ability to rapidly convert the exogenous phenol to tyrosine by tyrosine phenol lyase [34]. Benzoquinones are highly reactive, unstable and also toxic. Besides the purpose of chemical defense, *Tribolium* beetles and other insects use them also as tanning agent and to sclerotize cuticles [35]–[37], which requires perfect handling and detoxication systems. Thus understanding of the mechanisms involved in the autodetoxication of the defensive compounds might provide inspirations to manage this cosmopolitan pest and potentially other coleopteran pests. Obviously, tenebrionids are protected from their own toxic secretions by cuticular linings both internally and externally [5]. *Tribolium* beetles have the ability to partition the secretion away from the somatic cells, firstly by producing the secretions in the cuticle-lined organelles [14] and then keeping them in storage sacs (reservoirs) that are formed from invaginations of the cuticle [12]. The newly emerged *Tribolium* adults lack the defensive secretions, implying the need for building up an adequate self-protective barrier [8]. Consequently, if such self-protection systems could be broken, the pests will harm themselves. Here, we present the first transcriptome data on beetle stink glands, which allowed us to identify three genes necessary for the production of defensive quinones.

## Results

### Stink gland transcriptome sequencing

The odoriferous defensive stink glands were dissected and identified by their special morphological structures, vesicular organelles [13], [14] (***Figure S1***). mRNA sequencing was performed in six stink gland samples and one control sample (anterior abdomen, where no odoriferous glands are located) on a next generation sequencing (NGS) platform. The abbreviations for the samples were, s1: sample 1, anterior abdomen from wild-type; s2: prothoracic glands from *tar* mutant; s3: wild-type male prothoracic glands; s4: wild-type female prothoracic glands; s5: wild-type male abdominal glands; s6: wild-type female abdominal glands. After sequencing, 27.8 to 29.7 million reads were obtained from each sample. About 50% of them were successfully mapped to the reference database, i.e. mRNAs of the *Tribolium* official gene set (OGS) in Beetlebase [22], [38]. And the average depths/coverages were in the range of 23.5 to 27.8. Moreover, ratios of covered region in reference varied from 52.5% to 68.0%. Detailed statistics are presented in **Table 1**. The mapping of the different transcriptomics samples as individual tracks to the newest *Tribolium* genome assembly is provided by the public iBeetle Genome Browser (http://bioinf.uni-greifswald.de/gb2/gbrowse/tcas).

**Table 1:** Statistics of transcriptome sequencing.

Sample name	s1_ctl	s2_tthr	s3_mthr	s4_fthr	s5_mabd	s6_fabd
**Total reads**	29,527,715	29,690,989	28,544,764	29,350,110	27,929,437	27,786,784
**Mapped reads**	14,727,172	15,773,797	14,605,945	15,590,528	14,327,863	16,955,288
**Ratio of Mapping**	49.88%	53.13%	51.17%	53.12%	51.30%	61.02%
**Total mapped bases**	559,632,536	599,404,286	555,025,910	592,440,064	544,458,794	644,300,944
**Reference total base**	23,149,063	23,149,063	23,149,063	23,149,063	23,149,063	23,149,063
**Covered total base**	15,746,380	13,768,019	13,279,109	12,151,610	13,943,411	13,272,086
**Ratio of Covered region**	68.02%	59.48%	57.36%	52.49%	60.23%	57.33%
**Average Depth(Coverage)**	24.18	25.89	23.98	25.59	23.52	27.83
**Relative total reads**	1.00000	1.00553	0.96671	0.99399	0.94587	0.94104

s1_ctl: sample 1, anterior abdomen control; s2_tthr: sample 2, *tar* prothoracic glands; s3_mthr: sample 3, male prothoracic glands; s4_fthr: sample 4, female prothoracic glands; s5_mabd: sample 5, male abdominal glands; s6_fabd: sample 6, female abdominal glands.

### mRNA-seq library subtractions

**Figure 1:**
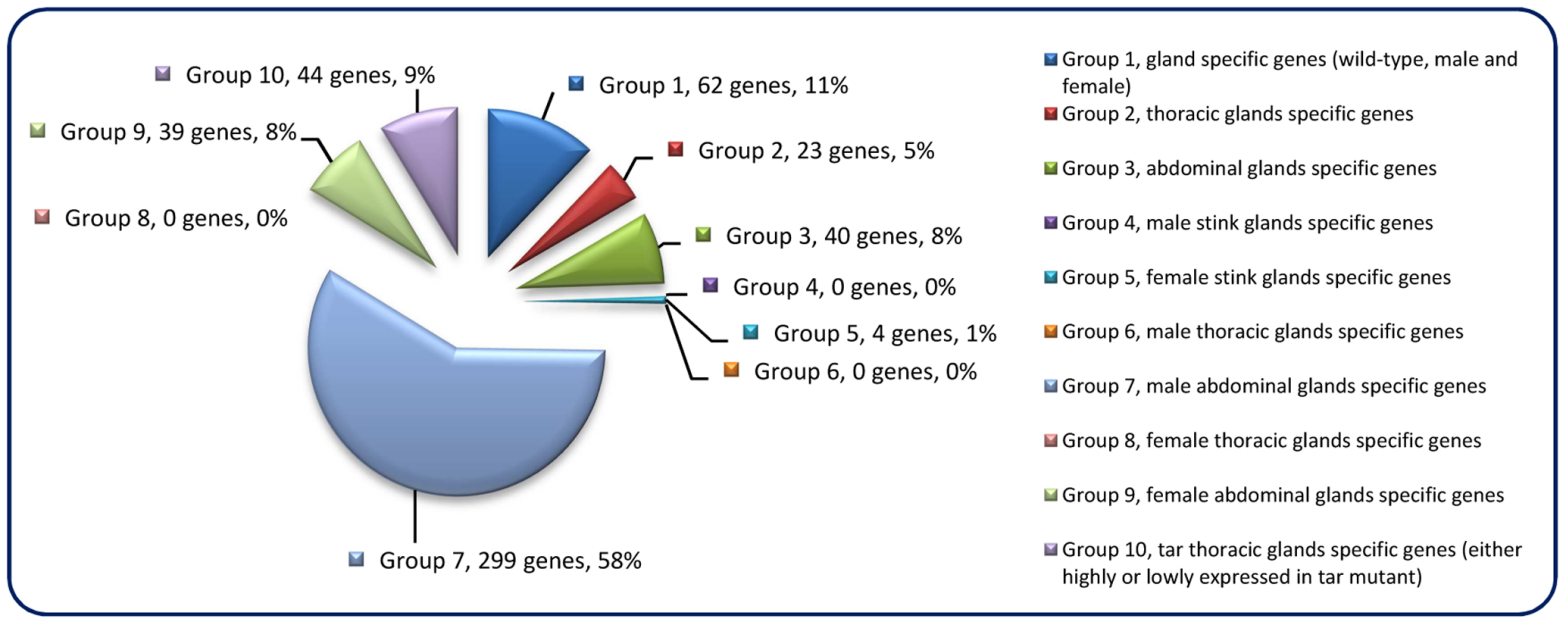
Odoriferous gland transcriptome screening result. Each pie slice is indicated with the group name, the number of genes classified, and their percentage. Each group is signified as a specific non-overlapping subtraction group with each gene belonging to only one group. E.g. Group 1 consists of genes that are highly expressed in all glands but not specifically in one gland type or only in one sex.

The constructed mRNA-seq libraries are presented in ***Dataset S1***, which shows the reads and coverage (depth) of each gene in all the samples, respectively. **Table 1** indicates that the relative total reads of all samples were quite close to each other. Therefore, the read number represented actual expression levels of all the *Tribolium* genes in various samples. In order to screen out the differentially or specifically expressed genes in odoriferous glands, ten different groups of subtractions were performed and in total 511 genes were identified (**Figure 1**; for detailed list and the subtractive conditions, see ***Dataset S2***). There were 62 genes in Group 1, standing for the gland-specific genes, which were highly expressed in all wild-type gland samples [s3, s4, s5, s6] but not in the anterior abdomen control [s1]. Group 2 presented 23 thoracic gland-specific genes, which had higher reads in wild-type thoracic glands [s3 and s4] than in wild-type abdominal glands [s5 and s6 (and s1 control)]. Group 3 had 40 abdominal gland-specific genes [s5 and s6 against s3, s4 (and s1)]. For sex related subtractions, Group 4 offered zero male stink gland-specific genes [s3 and s5 against s4, s6 (and s1)], but Group 5 had four female stink gland-specific genes [s4 and s6 against s3 and s5]. There were zero male thoracic gland-specific genes in Group 6 [s3 against s4 and s1], yet 299 male abdominal gland-specific genes in Group 7 [s5 against s6 and s1]. Meanwhile, there were also zero female thoracic gland-specific genes in Group 8 [s4 against s3 and s1], but 39 female abdominal gland-specific genes in Group 9 [s6 against s5 and s1]. And Group 10 presented 44 genes which were either up or down regulated in prothoracic glands of *tar* mutants. The high number of genes identified in Group 7 is probably due to the contamination of the abdominal gland sample by male accessory glands, which are hard to separate from the abdominal glands.

### Gene ontology annotation

**Figure 2:**
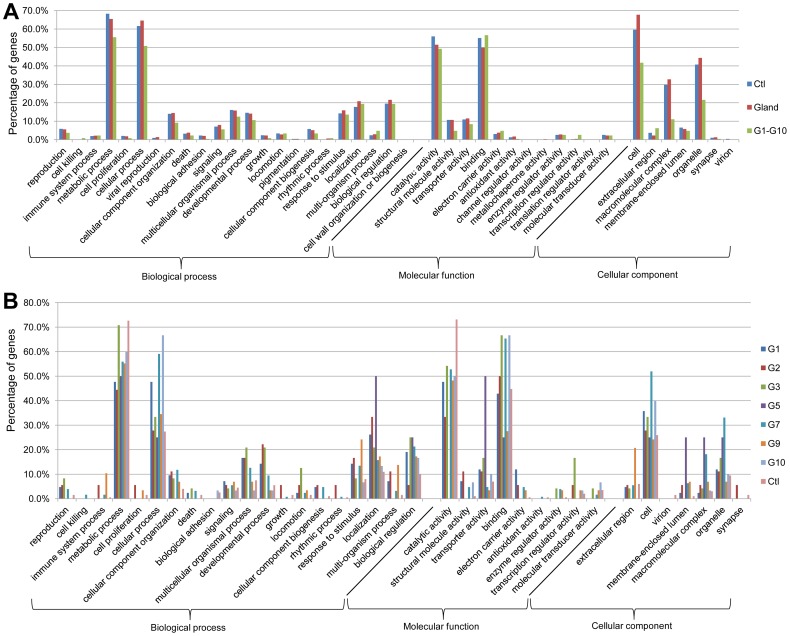
GO annotation of odoriferous glands transcriptome data. A, analyses of the genes abundant in control (Ctl; 1451 genes abundant in anterior abdomen transcriptome), wild-type glands (Glands; 1206 genes), and the genes identified in all ten subtraction groups (Figure 1) together (G1–G10; 511 genes). B, analyses of individual Group 1 to Group 10, respectively and the control (Ctl; 290 genes possessing at least 64 times higher reads in anterior abdomen than the wild-type gland samples). X-axis: different GO terms (level 2); Y-axis: percentage of the genes classified in each group.

Gene ontology (GO) annotation allows meta-analyses of gene populations and associates the targeted genes to specific terms with hierarchical vocabularies describing three independent ontologies: biological process, molecular function, and cellular component [39]. Analyses were performed with 1451 genes abundant in the control, 1206 genes abundant in wild-type glands, and the 511 genes from subtraction Group 1 to Group 10 (The genes are listed in ***Dataset S2***). Results (**Figure 2A**) showed that many genes were classified to metabolic and cellular processes in the GO term of biological process, and to catalytic activity and binding in molecular function. For the cellular component, most genes belonged to cell, macromolecular complex and organelle. These implied the existence of strong metabolisms in both gland and anterior abdomen. Moreover, similar trends were observed in separated analyses in the different subtraction groups (**Figure 2B**). Detailed GO results are presented in ***Dataset S3***.

### Transcriptomic exploration of candidate genes for quinone synthesis

**Figure 3:**
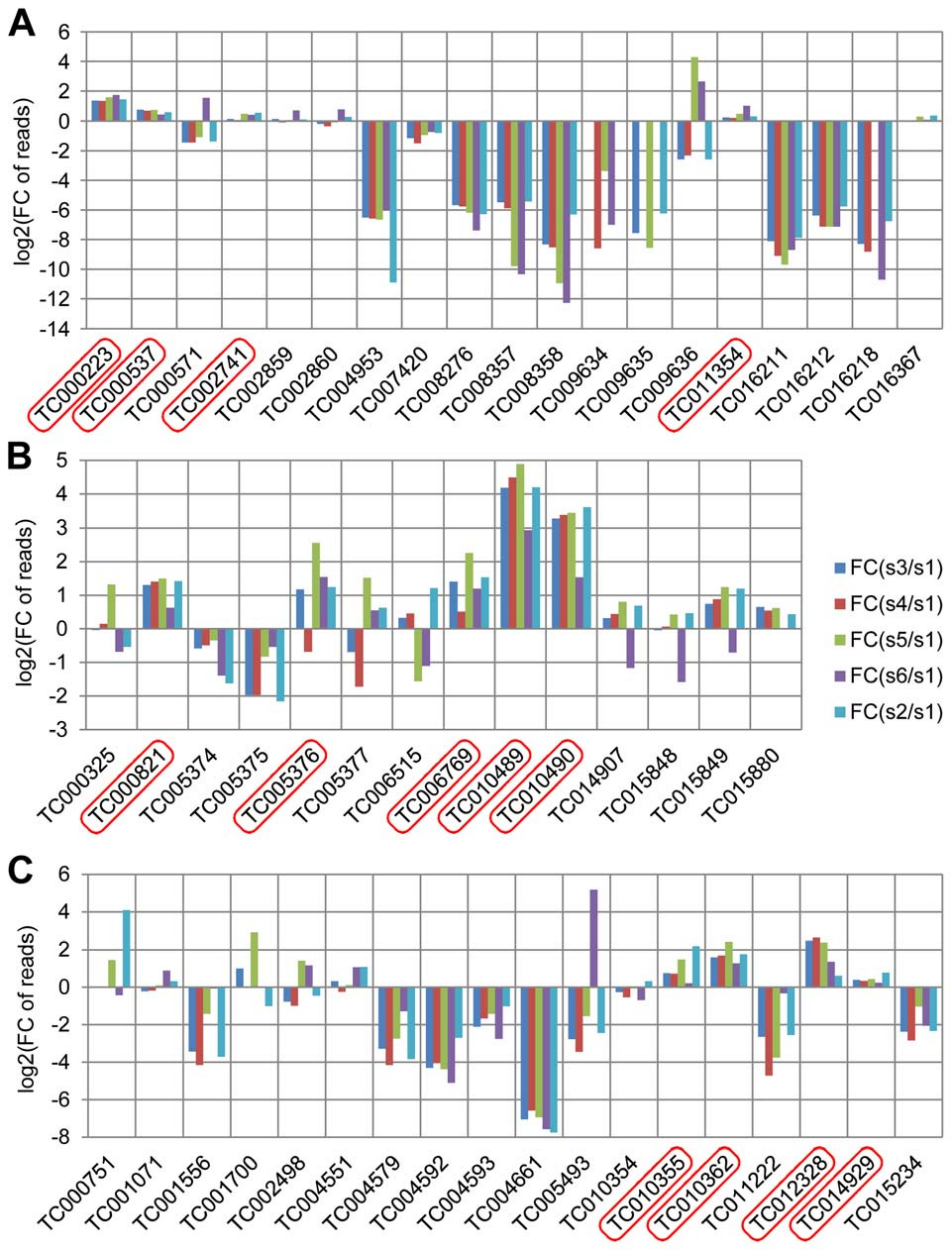
Annotated quinone synthesis-related genes and their relative gland transcriptome expression levels. A, glucosidases, 19 genes; B, phenol oxidases, 14 genes; C, peroxidases, 18 genes were annotated. In all charts, along the X-axes the different genes are presented, while the Y-axes present log2[fold change of reads in glands against control]. Abbreviations: FC: fold change; s1: sample 1, anterior abdomen as a control; s3: sample 3, male prothoracic glands; s4: sample 4, female prothoracic glands; s5: sample 5, male abdominal glands; s6: sample 6, female abdominal glands; s2: sample 2, *tar* prothoracic glands. The genes with higher reads in gland samples are marked with red squares.

Glucosidases, phenol oxidases, and peroxidases have been considered to be involved in the production of quinones in the odoriferous glands [14] and were annotated in the *Tribolium* genome [29]. In our stink gland transcriptome analysis, we have now explored these candidate genes for expression at the gland transcriptome level (**Figure 3**, details in ***Dataset S4***). In total, 19 glucosidase, 14 phenol oxidase, and 18 peroxidase encoding genes were identified through blast searches and conserved domain confirmation. Transcriptomic explorations revealed that at least four glucosidase (*TC000223*, *TC000537*, *TC002741*, and *TC011354*), five phenol oxidase (*TC000821*, *TC005376*, *TC006769*, *TC010489*, and *TC10490*) and four peroxidase (*TC010355*, *TC010362*, *TC012328* and *TC014929*) genes have increased reads in the gland samples, which confirms the importance of these three types of enzymes in defensive secretions and verifies the reliability of our transcriptome data.

### Functional analysis of the most highly and gland-specifically expressed genes

**Figure 4:**
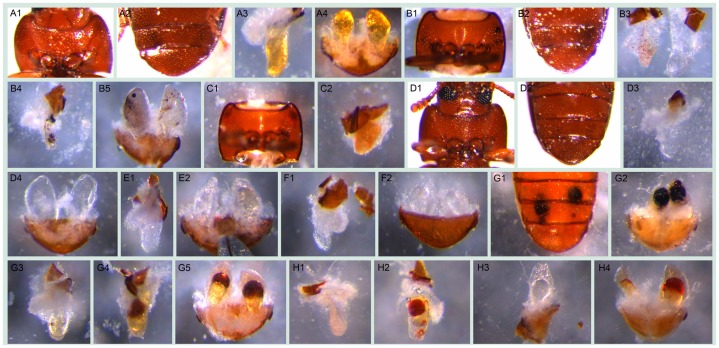
Visible morphological gland phenotypes after RNAi. A1–A4, wild-type: A1, thr (darker regions on two sides); A2, abd (darker regions on the tip); A3, thrg reservoir filled with yellowish oily fluid ; A4, abdg; B1–B5, *GT20* knock-down: B1, thr, visible melanized dots on both sides; B2, abd, darker gland regions; B3, thrg, left one, small localized brown dots, right one, tiny localized brown dots accompanied by colorless oily secretions; B4, thrg, localized melanized dots; B5, abdg, localized melanized (left) or brown (right) dots; C1–C2, *GT47* knock-down: C1, thr; C2, thrg, condensed secretions, i.e. not as semitransparent as in wild-type and close to solid; D1–D4, *GT62* knock-down: D1–D2, thr and abd with the gland regions almost invisible through cuticle; D3, thrg, very few colorless secretions, in some cases, empty-looking (not shown); D4, abdg, colorless secretions; E1–E2, *GT39* knock-down, thrg (E1) and abdg (E2), colorless secretions; F1–F2, *GT63* knock-down, thrg (F1) and abdg (F2, ventral view), colorless secretions; G1–G5, *GT02* knock-down, at stage A10 localized melanized dots (G1, abd; G2, abdg), at stage 24 localized brown dots to different extent (G3 and G4, thrg, G5, abdg); H1–H4, *GT25* knock-down, various phenotypes: H1 and H2, thrg, tiny (H1) or big (H2) localized brown dots; H3, left half abdg, oily colorless secretions; H4, abdg, localized brown dots. The beetles used for G3–G5 were from stage A24, all the others were at stage A10. The statistics of the visible phenotypes are in Figure 5B. Abbreviations used in this legend: stage A10, adults ten days; A24, twenty four days after eclosure; abd, abdominal tip, ventral view; abdg, dissected abdominal tip with abdominal glands, dorsal view; thr, prothorax, ventral view; thrg, dissected prothoracic gland(s), with the opening on top, the secretory cells are attached to the reservoir.

**Figure 5:**
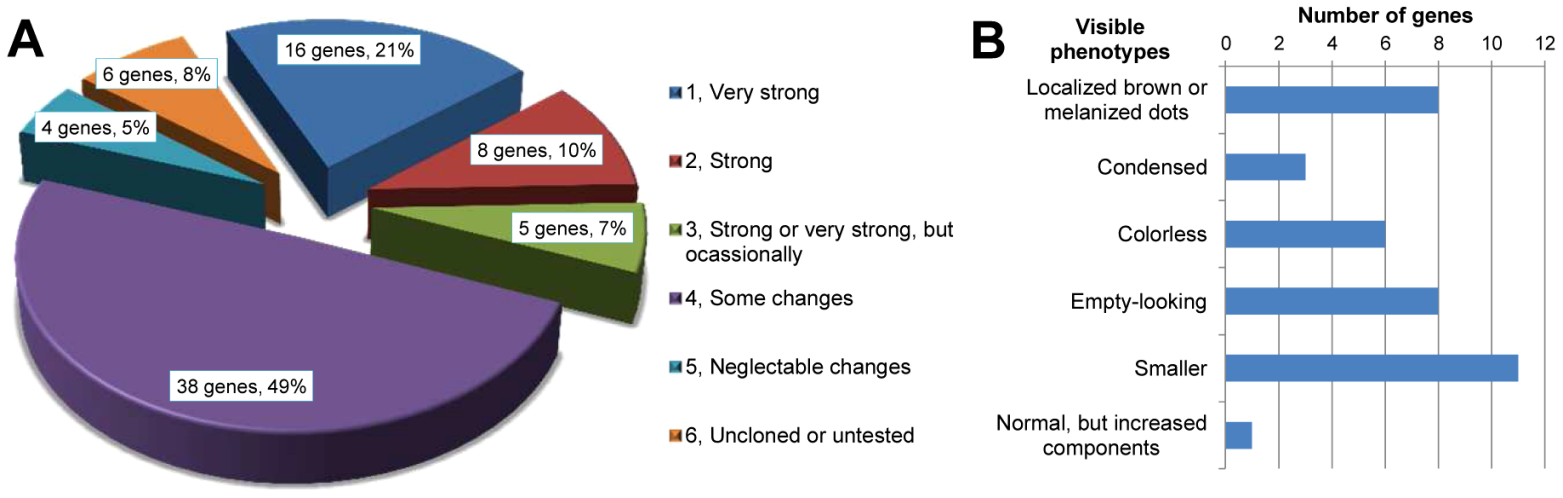
Phenotype classifications of 77 highly gland-specifically expressed genes by RNAi. A, Description of phenotype strengths: 1, Very strong: at least one type of chemical was undetectable or less than 5% left in thoracic or/and abdominal glands; 2, Strong: at least one type of chemical was 75%–95% reduced or increased by more than 75% in thoracic or/and abdominal glands; 3, Strong or very strong but occasionally: phenotype was similar to 1 or 2 but not observed in all the injected beetles; 4, Some changes: at least one type of chemical was 25%–75% reduced or increased in thoracic or/and abdominal glands; 5, Neglectable changes: less than 25% reduction or increase in any type of chemical; 6, uncloned or untested. B, Strong and very strong gland phenotypes (strengths 1–3 in panel A) in details, some genes had more than one phenotype. Except for the last two classifications, examples are provided in Figure 4: localized brown or melanized dots (4B1-B5 and G2-G5); condensed (4C1-C2); colorless (4E1-F2); empty-looking (4D3).

In order to find novel gene functions involved in quinone synthesis, we functionally analyzed 77 genes from transcriptomic subtraction groups 1, 2, and 10 that were at least 64× higher expressed in the glands compared to the control tissue. RNAi of these genes resulted in various abnormal visible phenotypes (**Figure 4**). Additionally, gas chromatography–mass spectrometry (GC-MS) measurements revealed the alterations of different chemical components in both pairs of glands (an example of the chromatogram is depicted in ***Figure S2***). The main components identified are listed in **Table 2**. Based on the extents of alterations of the chemicals, phenotypes were classified into six strengths (**Figure 5A**). 29 genes (38%, strength 1–3) showed strong changes, i.e. more than 75% reduction of at least one component, which were mostly accompanied with visible phenotypes (**Figure 5B**). In total, 67 of 77 genes (87%) showed alterations of at least one secreted chemical. Detailed descriptions on the phenotypic changes of all the 77 genes can be found in ***Dataset S5***. In addition, gland cellular morphology was explored in all the 29 genes with strong phenotypes, but no visible abnormalities were observed in the secretory cells (data not shown).

**Table 2:** Main gland volatiles identified by GC-MS.

Retention indices	Compound name	CAS-Number	MW	Comment
1011/1018	methyl-1,4-benzoquinone	000553-97-9	122.04	
1098/1109	ethyl-1,4-benzoquinone	004754-26-1	136.05	
1350/1367	methyl-1,4-hydroquinone	000095-71-6	124.05	
1432	ethyl-1,4-hydroquinone	2349-70-4	138.07	
1477	1,6-pentadecadiene	58045-15-1	208.38	a, low peak
1492	1-pentadecene	013360-61-7	210.24	
1552	1,2-dimethoxy-4-n-propylbenzene	005888-52-8	180.12	b, low peak
1593	1-hexadecene	000629-73-2	224.25	low peak
1663/1672	1,8-heptadecadiene	Not available	236.25	a
1693	1-heptadecene	006765-39-5	238.27	

a: based on previous data [21], the positions of the double bonds (especially the second one) need to be confirmed; b: based on PBM (probability-based matching algorithm) database searching result (matching quality 86%). The data refer to both prothoracic and abdominal glands from wild-type adult beetles at stage A10.

### Quantification of volatile gland contents

**Figure 6:**
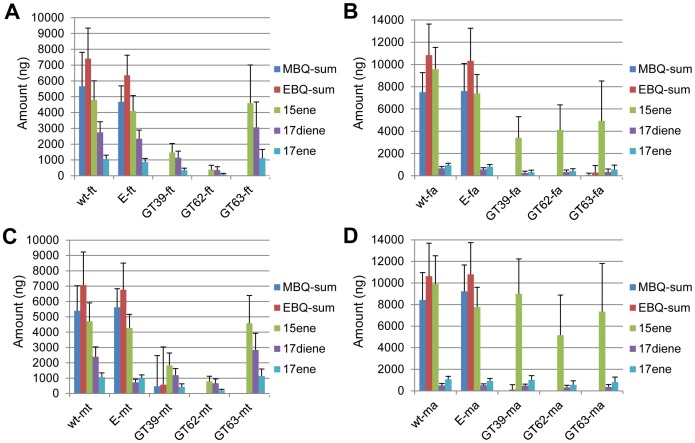
Quantification of main volatile glandular chemicals by GC-MS in wild-type and novel *quinone-less* gene RNAi-knock-downs. Comparisons in female thoracic glands (ft) (A), female abdominal glands (fa) (B), male thoracic glands (mt) (C), and male abdominal glands (ma) (D). Y-axis: amount in nanogram; X-axis: wild-type and different RNAi-knock-downs. Abbreviations: wt: wild-type; E: dsEGFP-injected control; GT39: *Tcas-ql VTGl*; GT62: *Tcas-ql ARSB*; GT63: *Tcas-ql MRP*; MBQ-sum: methyl-1,4-benzoquinone; EBQ-sum: ethyl-1,4-benzoquinone; 15ene: 1-pentadecene; 17diene: 1,8-heptadecadiene; 17ene: 1-heptadecene. The error bars indicate standard deviations at N = 15–30.

Previous research has revealed the amount of different glandular components only on the whole beetle level [8], [9], [16], [18], [40]–[43]. In order to elucidate the chemical compositions of the volatiles in the different pairs of glands and the extent of reduction after gene knock-downs, three genes with strong quinone-less phenotypes were chosen from the 77 tested genes to quantify different glandular components. Wild-type and Enhanced green fluorescent protein (EGFP) double stranded RNA (dsRNA)-injected beetles were used as controls. **Figure 6** shows the complete losses of all quinones in both pairs of glands in both females and males from the knock-down beetles of these three *quinone-less* genes (except for one *GT39* dsRNA injected male out of sixteen injected males). In comparison, the alkenes were reduced to different extents. Statistical analyses revealed significant differences between wild-type and the knock-downs (***Dataset S6***), EGFP dsRNA injection surprisingly caused a few significant differences in alkenes compared to wild-type but not in quinones. Interestingly, all the alkenes in prothoracic glands of *GT63* knock-downs were not statistically different from the wild-type, while only heptadecadiene and heptadecene in the abdominal glands showed the same trend. Sex differences were also analyzed (***Dataset S6***), which showed that most chemicals had no significant differences between males and females, except for all the alkenes in abdominal glands of *GT39* knock-downs and in thoracic glands of *GT62*. In wild-type, only heptadecadiene showed a significant difference between different sexes while the other chemicals did not.

Additionally, the amount of all the main components in wild-type A10 beetles is presented in **Table 3** (details in ***Dataset S6***). The prothoracic glands possess about 40% of either quinones or alkenes of all the stored secretions in the whole beetle, while abdominal glands have about 60%. But the molar ratios of quinones to alkenes are almost the same in both pairs of glands (thr, 2.60; abd, 2.70–2.74). And the molar ratios of MBQ to EBQ vary from 0.77 to 0.88 in different gland and sex levels. The only major dissimilarity between those two glands is the composition of distinct alkenes. The prothoracic glands have higher portions of 1-heptadecene (17ene) and 1,8-heptadecadiene (17diene), especially the former, but a lower portion of 1-pentadecene (15ene) (15ene: 17diene: 17ene = ∼60%: 28%: 12% in thr, ∼88%: 4%: 8% in abd).

**Table 3:** Quantification of the main volatiles in wild-type odoriferous stink glands.

Sex & gland types	MBQ (µg)	EBQ (µg)	15ene (µg)	17diene (µg)	17ene (µg)	Quinones (nmol)	Alkenes (nmol)
**male thr**	5.39±1.63	7.08±2.15	4.71±1.2	2.39±0.64	1.08±0.27	96.21±28.97	37.06±9.44
**fem. thr**	5.66±2.15	7.41±1.93	4.8±1.21	2.74±0.68	1.06±0.25	100.81±29.82	38.84±9.54
**male abd**	8.43±2.53	10.63±3.05	9.91±2.61	0.51±0.17	1.06±0.28	147.18±42.85	53.74±14.1
**fem. abd**	7.51±1.75	10.84±2.79	9.58±1.95	0.66±0.16	0.94±0.18	141.24±33.39	52.32±10.32
**male thr+abd**	13.82±3.61	17.71±4.6	14.62±3.17	2.9±0.71	2.14±0.46	243.39±62.9	90.8±19.16
**fem. thr+abd**	13.2±3.18	18.25±4.17	14.31±2.65	3.36±0.73	1.97±0.34	242.34±54.2	90.58±16.24

The amounts are indicated as mean±standard deviation. N = 15–30. Abbreviations: fem.: female; thr: thoracic glands; abd: abdominal glands.

Full length cDNAs of the three *quinone-less* genes, *GT39*, *GT62* and *GT63*, were cloned and the sequences submitted to GenBank (accession numbers JX569829, JX569830, and JX569831, respectively). Based on the phenotypes and their homology (see ***Dataset S7, S8, and S9*** for respective protein sequences), *GT39* has been designated as *Tcas-quinone-less vitellogenin-like* (*Tcas-ql VTGl*), *GT62* as *Tcas-quinone-less arylsulfatase b* (*Tcas-ql ARSB*) and *GT63* as *Tcas-quinone-less multi-drug resistance protein* (*Tcas-ql MRP*).

### Phylogeny of the three newly identified quinone-less proteins

**Figure 7:**
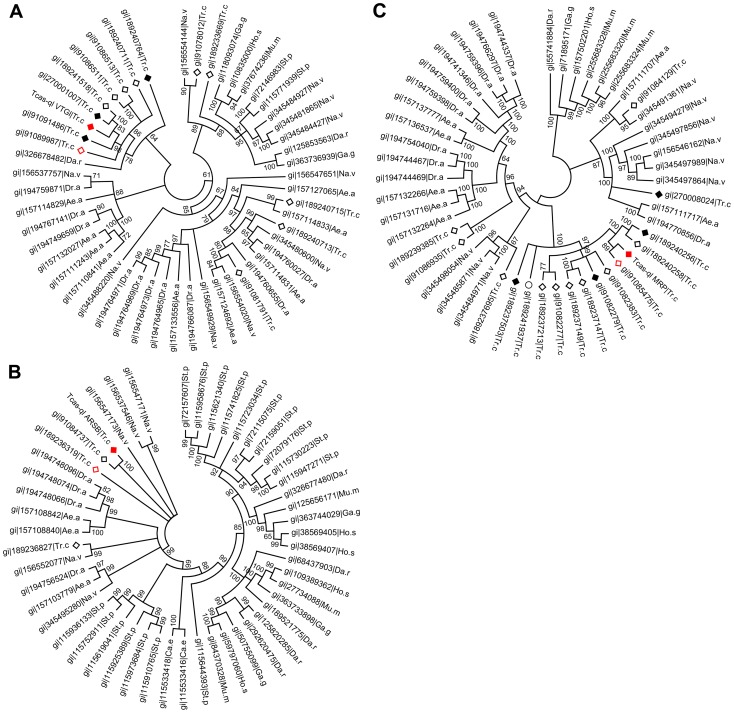
Phylogenetic trees of homologs of the three novel quinone-less proteins. A, Tcas-ql VTGl (GT39); B, Tcas-ql ARSB (GT62); C, Tcas-ql MRP (GT63). All the *Tribolium* homologs were marked based on their relative gland expression levels (Figure 8). The solid red squares indicate each of the three *quinone-less* gene encoded proteins, the open red squares relatively high expression in gland samples, the solid black squares relatively high expression in the control sample, the open black squares the other *Tribolium* homologs, and an open black circle indicates a *Tribolium* homolog without OGS annotation. The numbers on the branching points are the statistical frequencies. Abbreviations of species names are: Ae.a, *Aedes aegypti*; Ca.e, *Caenorhabditis elegans*; Da.r, *Danio rerio*; Dr.a, *Drosophila ananassae*; Ga.g, *Gallus gallus*; Ho.s, *Homo sapiens*; Mu.m, *Mus musculus*; Na.v, *Nasonia vitripennis*; St.p, *Strongylocentrotus purpuratus*; Tr.c, *Tribolium castaneum*.

**Figure 8:**
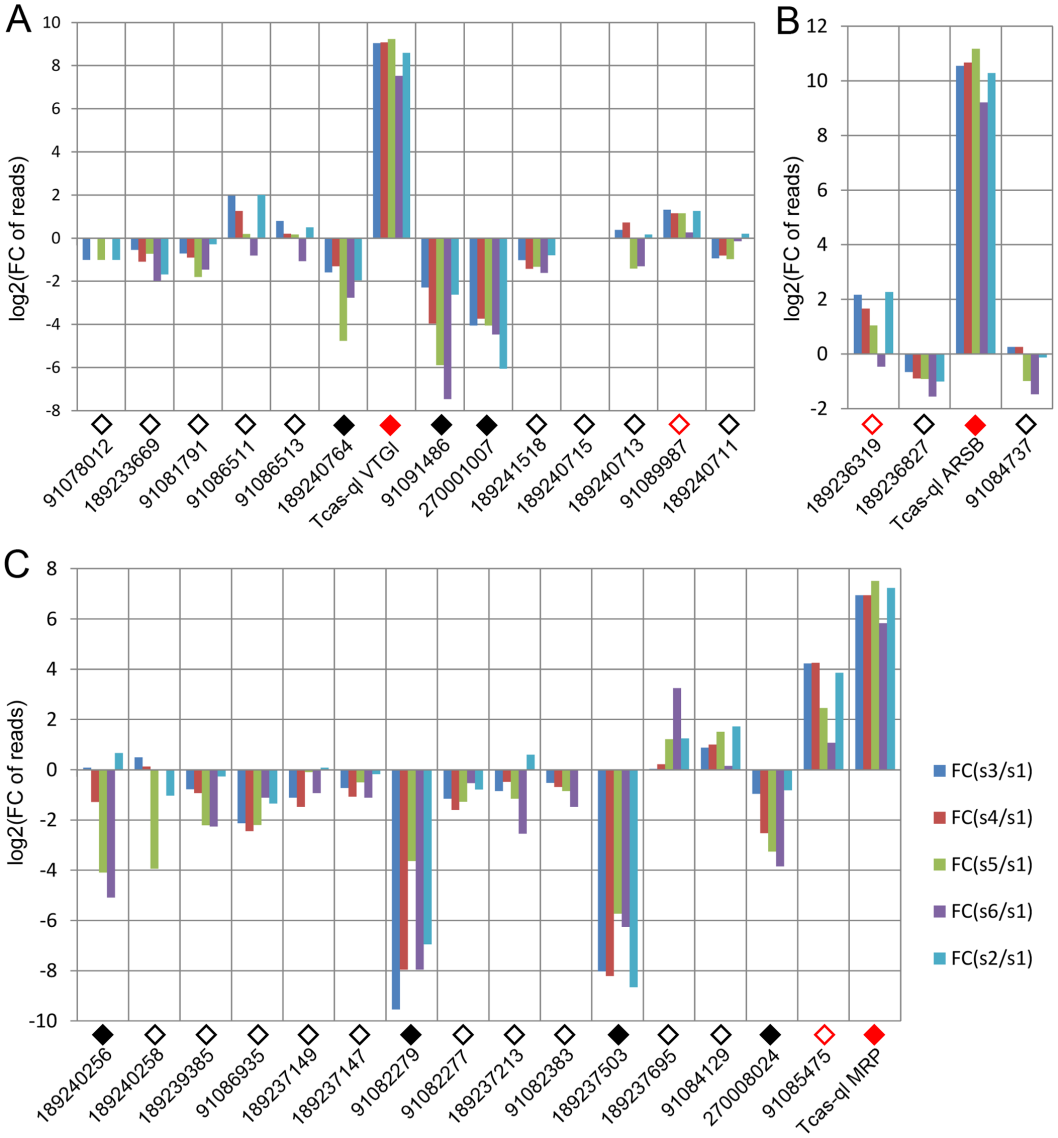
Relative transcriptomic gland expression levels of the *Tribolium* homologs of the three novel *quinone-less* genes. A, *Tcas-ql VTGl* (*GT39*) homologs. B, *Tcas-ql ARSB* (*GT62*) homologs. C, *Tcas-ql MRP* (*GT63*) homologs. In all charts, along the X-axes the different genes are presented (using the same GI numbers and expression level square codes as in Figure 7), while Y-axes present log2[fold change of reads in glands against control]. For abbreviations see Figure 3.

After quantification of the gland volatiles in these three gene knock-downs, their phylogeny was explored. In the phylogenetic tree (**Figure 7A**), Tcas-ql VTGl (GT39) was classified together with eight other *Tribolium* homologs, and 11 homologs were grouped in a branch close by, including proteins from *Strongylocentrotus purpuratus, Danio rerio, Gallus gallus, Homo sapiens, Mus musculus*, and *Nasonia vitripennis* (see ***Dataset S7*** for all the sequences). Tcas-ql ARSB (GT62) was grouped together with two other *Tribolium* homologs (**Figure 7B**, see ***Dataset S8*** for all the sequences), which were closest related to three *Nasonia* proteins, similarly to Tcas-ql MRP (GT63, **Figure 7C**, see ***Dataset S9*** for all the sequences). Since all three *quinone-less* gene encoded proteins had several *Tribolium* homologs, we checked their homologs expression levels in the gland tanscriptome. The homologs were linked with corresponding GLEAN predictions and explored in the transcriptomic libraries (***Dataset S10***). It was shown (**Figure 8**) that no other gene was as highly expressed in the gland samples except for the three identified *quinone-less* genes, although some genes contained more than 16 times (2 to the power of 4, GI:91085475, **Figure 8C**) or 4 times (2 to the power of 2, GI:189236319, **Figure 8B**) higher reads in some cases. In conclusion, this indicates that all three *quinone-less* genes most probably have evolved independently and specifically for the quinone-based chemical defensive system in *Tribolium*.

### Expression patterns of the *quinone-less* genes in gland tissue

**Figure 9:**
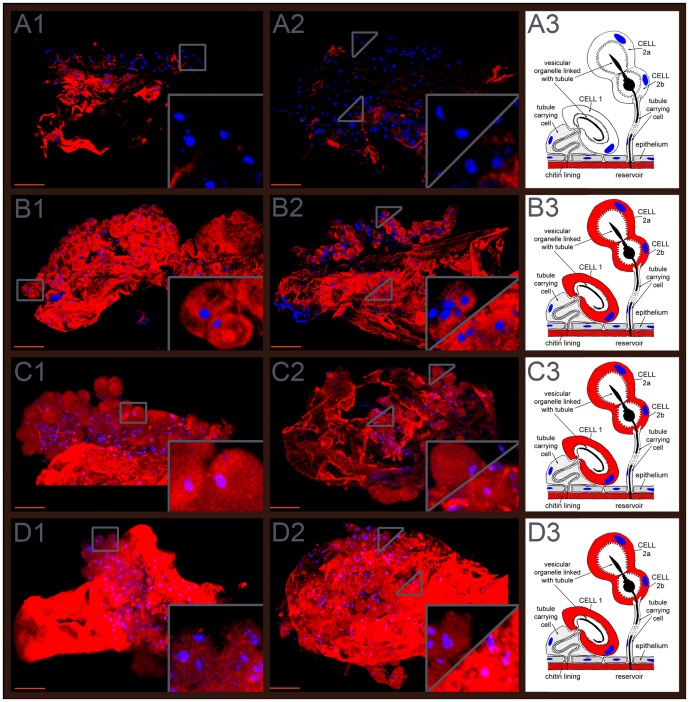
Expression patterns of the three *quinone-less* genes. The first column (A1, B1, C1, and D1) shows prothoracic glands, the second (A2, B2, C2, D2) abdominal glands, and the third (A3, B3, C3, D3) gland schemes drawn based on own observations and previous depictions of Happ [14] and Eisner [13]. A1–A3, *Tcas-ql VTGl* (*GT39*) sense probes. B1–B3, *Tcas-ql VTGl* (*GT39*) antisense probes. C1–C3, *Tcas-ql ARSB* (*GT62*) antisense probes. D1–D3, *Tcas-ql MRP* (*GT63*) antisense probes. In the right lower corner of the panels in the first and second column, the expression was digitally magnified 4 times, with its original positions indicated by a square box or triangles within the same panel. Cell type 1 and cell type 2 were separately indicated in the abdominal glands, cell type 1 was zoomed in the right lower corner of the square, and cell type 2 in the left upper part of the square. Scale bars: 50 µm.

The expression patterns of *Tcas-ql VTGl* (*GT39*), *Tcas-ql ARSB* (*GT62*) and *Tcas-ql MRP* (*GT63*) were explored in dissected odoriferous glands by gland whole mount fluorescent *in situ* hybridization (GWMFISH). All three genes showed strong expressions in both gland cell type 1 and cell type 2 [13], [14] of both pairs of glands (**Figure 9**), which confirms their involvement in *Tribolium* defensive secretion.

### Microbe inhibition assays

**Figure 10:**
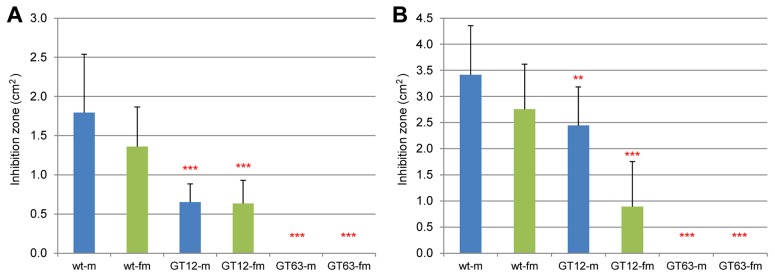
Microbe growth inhibition assays of wild-type and RNAi-knock-down glands. The fungus *A. niger* (A) and the bacterium *A. globiformis* (B) were analyzed for gland-mediated growth inhibition. Y-axes indicate the areas of respective inhibition zones (cm^2^). X-axes: sex-specific wild-type (wt) and different RNAi-knock-downs (m: male; fm: female; GT12: gene causing alkene-less phenotype; GT63: *Tcas-ql MRP*). Non-parametric comparisons were made between wild-type and knock-downs using Wilcoxon method, ***, P<0.001; **, 0.001<p<0.01. The error bars indicate standard deviations at N = 11–27.

Microbe inhibition tests were carried out to identify the effect of losing quinones. It had been shown that beetle chemical secretions – especially the benzoquinones – can inhibit the growth of several microbes common to flour with artificial MBQ having the same effect [44], [45]. In our experiments, a fungus, *Aspergillus niger*, and a gram positive bacterium, *Arthrobacter globiformis*, were used to test the strength of the chemical defense mediated by beetle glands. As a control, we used RNAi against *GT12* (detailed analysis on this gene will be presented elsewhere), representing a gene causing an alkene-less phenotype at knock-down, which is accompanied by only a slight reduction of quinones (see ***Dataset S5***). The results (**Figure 10**) showed that wild-type beetle secretions could inhibit microbe growth in a certain area, but loss of quinones led to undetectable inhibitions (*GT63*), while an alkene-less state had only reduced inhibition effects (*GT12*).

### Phenol oxidase activity assays

**Figure 11:**
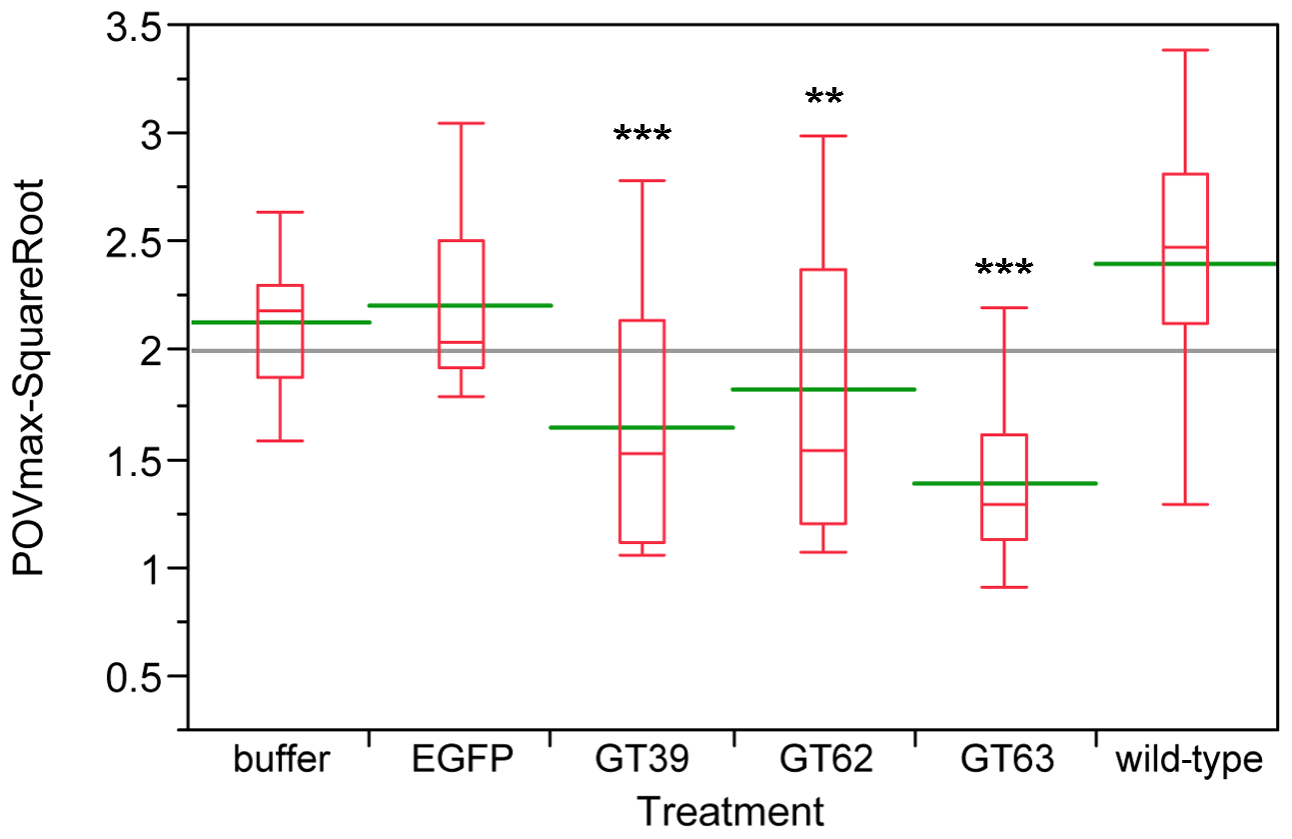
Phenol oxidase (PO) activity assays of wild-type and novel *quinone-less* gene RNAi knock-downs in females. The Y-axis indicates the square root of PO Vmax, red boxes are boxplots, green lines represent the mean value, the gray line represents the grand mean, while the X-axis presents wild-type, control injections, and different RNAi-knock-downs (N = 12–25). Buffer: buffer-injection control; EGFP: dsEGFP-injection control; GT39: *Tcas-ql VTGl*; GT62: *Tcas-ql ARSB*; GT63: *Tcas-ql MRP*. The asterisks (*) mark the t-test results comparing to wild-type: ***: p<0.001; **: 0.001<p<0.01. Buffer- and EGFP-injected controls were not significantly different from wild-type.

Since chemical defense systems are responsible for defending the host from infection, we wanted to test, whether also a part of the innate immune system is affected directly or indirectly by the quinone-less phenotype. Thus, after RNAi-mediated knock-down of the *quinone-less* genes, phenol oxidase (PO) activities were measured as a general index of the melanization innate immune responses in invertebrates [46]. Compared to wild-type beetles, the three *quinone-less* gene knock-downs had significantly reduced levels of PO-activity both in females (**Figure 11**) and males (***Figure S3***), while buffer or EGFP dsRNA injected animals did not show significant changes. These data indicate that the extra-corporal chemical defense may be linked with the function of a part of the innate immune system in *Tribolium*.

## Discussion

Transcriptome sequencing was performed in odoriferous gland samples from *Tribolium castaneum* to identify novel gene functions in defensive quinone synthesis. In a next generation sequencing approach, 27.8–29.7 million reads were obtained, of which only 50 to 61% reads were successfully mapped to the *Tribolium* genome. Part of the low mapping ratio is probably explainable by the strain differences used for genome sequencing (inbred Georgia 2 strain) [29] and the San Bernardino strain we used for transcriptomics together with the strict mapping parameters. However, this did neither affect the comparison and subtraction between different samples, nor the library subtraction results, since all our wild-type samples have the same strain background and all samples were treated identically. Only the *tar* mutation is in different genetic background derived from *sooty* [47] and *Maxillopedia-Dachs3* strains (*mxp^Dachs3^*) [31]. However, the mapping ratio for this sample was similar to the others. Another reason for the low mapping ratio might be that the official gene set was from a combination of different automated gene prediction programs and annotation pipelines [29], which makes it possible that there were still unidentified genes in the *Tribolium* genome.

The comparisons we performed were between different tissues in wild-type and *tar* mutant. During the library subtractions, we chose a general cut-off of fold change at 64 times, which is much higher compared to many microarray analyses (mostly two times), but the number of the genes we got was reasonably high and seemed suitable to start to work with. Only subtraction Group 7 (male abdominal gland-specific genes), with 299 genes (**Figure 1**), showed an unusual high number, which might, however, be explained by the fact that the male accessory glands are hard to dissect away from the abdominal glands and many of the genes in Group 7 might actually be male accessory gland-specifically expressed genes. As these were not a topic of this study, these potentially interesting genes remain for future analysis.

GO annotations revealed that the glands have quite active metabolisms with many catalytic and binding related genes being highly expressed. The GO annotation rate of the genes, that had coverage of more than 50 in all the glands, was 70.1%, while the control had 78.8% (***Dataset S3***). This suggests that there are more orphan genes expressed in *Tribolium* odoriferous glands than in the control. In addition, only 53.6% of all the 511 genes from the subtractions were annotated, and surprisingly, Group 7 (male specific glands genes) had an annotation rate of only 42.5%, suggesting an even higher number of genes with unknown functions. We identified also some glucosidases, phenol oxidases and peroxidases highly transcribed in the glands, that are candidate enzymes to be involved in quinone biosynthesis. In conclusion, our transcriptome data have reliably detected candidate genes involved in quinone biosynthetic mechanisms of chemical defense in the red flour beetle.

Exploring the functions of a first batch of highly gland-specifically expressed genes by RNAi and GC-MS to potentially identify novel gene functions in quinone biosynthesis, 67 of 77 genes (87%) showed alterations of at least one secreted chemical, which not only confirmed their importance for semiochemical synthesis, but also signified the effectiveness of our transcriptome screening. Surprisingly, some genes with very high reads in the glands showed no big changes at the chemical level. For example, *GT26* (*TC007317*), *GT35* (*TC010551*) and *GT41* (*TC011337*) from Group 1 had more than 441, 514 and 690 times higher reads respectively in all wild-type gland samples than control (***Dataset S1***), however, their knock-downs showed less than 75% reductions, or even neglectable changes (***Dataset S5***). We suggest that these genes might be involved in other biological processes indirectly related to chemical secretion. Additionally, in Group 10, the *GT23* gene (*TC006131*) that had more than 126 times enriched reads in s2-tthr (*tar* prothoracic gland sample) compared to s3-mthr and s4-fthr (male and female prothoracic gland samples; ***Dataset S1***), showed no changes in prothoracic glands, but slightly increased amounts of quinones and reduced amounts of alkenes in abdominal glands. Encoding the odorant binding protein 21 (OBP21, GI:270012767), GT23 might be involved in olfaction system. It is possible that the mutated *tar* somehow caused the mis-expression of this gene in a different type of tissue, or OBP21 belongs to the ubiquitous OBP type, such as encapsulin [48], which is probably involved in diverse physiological functions [49]. Moreover, *GT23* showed about 10 times more reads in wild-type prothoracic glands than in the control sample. In addition, another OBP (*GT76*) and two chemosensory protein (*GT30*, *GT77*) encoding genes showed remarkable expression changes in the prothoracic glands of wild-type compared to *tar* mutants in the opposite direction. *GT30*, *GT76*, and *GT77* are expressed at high levels in wild-type prothoracic glands, but their expression is strongly reduced in *tar* mutants. None of these four genes is expressed at significant levels in the abdominal glands, indicating a specific function for the anterior glands. However, no significant changes in volatile gland contents could be detected after RNAi knock-down of those genes.

During the morphological analyses, many abnormal glands were observed (**Figure 4**). However, besides the quantitative changes in the main glandular volatiles, GC-MS of the referred gene knock-downs showed no additional peak compared to wild-type, which indicates non-volatility of the accumulated brownish or black substances. The gland phenotypes could be explained as the knock-downs triggered the inhibition of chemical syntheses or the blocking of their transportation, or in some cases, the accumulation of intermediate substrates or unknown brown or black polymers. It was proposed for previously identified *msg* mutants that the black material is of high-molecular-weight and polymeric consisting of polymerized prematurely formed quinones due to the absence of the inhibitor in oxidation of hydroquinone [30], [50]. Moreover, the lack of specific amphipathic molecules – such as terminal alkenes – might cause the various phases (probably organic and aqueous) seen e.g. in **Figure 4** **G4, G5**.

In the quantification part, the main glandular contents were quantified separately in both pairs of glands for the first time. Assuming that stage A10 at 32.5°C is equal to A12 at 30°C, which was predicted based on the *Tribolium* life parameter table [51], the MBQ and EBQ amounts we got (***Dataset S6***) in wild-type were 20–30% (females) and 40–70% (males) higher than the amounts detected by Unruh *et al.* [8]. This indicates that the dissection based extraction is much more accurate than the homogenization based method, since the latter may cause the loss of unstable chemicals during the crude preparation. Moreover, in our experiments, the males and females were not separated before harvesting, which is much closer to the natural conditions compared to the method Unruh *et al.* used [8]. In addition, more EBQ was detected in our tests (molar ratio of MBQ/EBQ: 0.81 in female, 0.87 in male), while the previously reported ratio was in the range of 0.59–0.61 [18], [42]. However, the weight ratio of quinones in the whole beetle (61.5%) was only a bit higher than 58.3% reported previously [18]. Interestingly, different hydrocarbon compositions were observed between the prothoracic and abdominal glands, which might reflect the dissimilar usage of their precursors, fatty acids [52], in distinct body parts and sexes. Furthermore, except for heptadecadiene in abdominal glands, all other components presented no significant differences between male and female at stage A10. Therefore, we propose that both sexes possess similar secretion levels in normal environment, and the higher level of benzoquinones in female observed before [8] was due to a different energy allocation when they produce no or less eggs as virgins, since reproduction (mating and egg production) could change the energy allocation and fitness in several other species [53]–[57].

After these first functional analyses of the complete set of highly gland-specifically expressed genes, three genes causing a quinone-less phenotype at knock-down were characterized further in our work. The quantitative data clearly showed the loss of quinones and the reduction of alkenes in both pairs of glands. Surprisingly, all the alkenes in *Tcas-ql MRP* (*GT63*) knock-downs showed no significant differences to wild-type in prothoracic glands but were significantly different in abdominal glands, while *Tcas-ql VTGl* (*GT39*) knock-downs caused significant differences to wild-type in almost all the comparisons except the three alkenes in the male abdominal glands. *Tcas-ql ARSB* (*GT62*) knock-downs showed significant changes to wild-type in all comparisons (***Dataset S6***). This implies that *Tcas-ql MRP* (*GT63*) has distinct functions in different glands, while *Tcas-ql VTGl* (*GT39*) and *Tcas-ql ARSB* (*GT62*) function diversely in both glands of the distinct sexes.

Potential molecular functions of these three genes in quinone synthesis are predicted on account of the homology and conserved domain analyses. Possessing a pancreatic lipase-like enzyme domain, Tcas-ql VTGl (GT39) is a vitellogenin like protein (31% identity). Vitellogenin is classified as a glycolipoprotein, having properties of a sugar, fat and protein, and belongs to a lipid transport protein family [58]. Some data in mealworm showed that a vitellogenin like protein (19.6% identity) could enhance the melanin synthetic process [59], in which o-quinones were produced in an intermediate step right before synthesizing eumelanin [60], [61]. Possibly, the benzoquinone production in odoriferous glands has similar pathways, in which Tcas-ql VTGl (GT39) regulates the related enzyme activity or reactions yet with more important roles, since almost no quinones were detected in its knock-down. Provided that common synthetic steps exist, the black material in *msg* and *tar* mutants might actually be composed of melanin or something alike.

Tcas-ql ARSB (GT62) is an arylsulfatase B (ARSB) protein (45% identity), which has a sulfatase domain responsible to hydrolyze sulfates in the body by breaking down large sugar molecules called glycosaminoglycans (GAGs). ARSB targets two GAGs in particular: dermatan sulfate and chondroitin sulfate. ARSB is located in lysosomes, compartments within cells that digest and recycle different types of molecules [62]. The deficiency of ARSB is the cause of mucopolysaccharidosis VI (MPS VI) which occurs in humans and cats, called also Maroteaux-Lamy syndrome. MPS VI is a progressive condition that causes many tissues and organs to enlarge and become inflamed or scarred, skeletal abnormalities are also common in this condition [63], [64]. Since lysosomes are the waste disposal system in the cell [65], a few possible functions of Tcas-ql ARSB (GT62) are proposed. Firstly, Tcas-ql ARSB (GT62) may have important roles in the detoxication of toxic substances in gland cells, whose knock-down leads to the initiation of an assumed feedback loop, then to the inhibitions of secretory chemical syntheses. Secondly, since different subcellular localization prediction tools gave distinct results (data not shown), Tcas-ql ARSB (GT62) may not be located in lysosomes but in the cytoplasm or elsewhere, functioning as an essential transporter for the intermediates involved in both quinone and alkene production, or as a key enzyme responsible for the activation of the newly translated transporters or other vital related proteins, or simply controlling the energy source of transportation, such as the pH gradient, or ion donators.

Tcas-ql MRP (GT63) belongs to ATP-binding cassette transporters, subfamily C (ABCC), which is also known as multidrug resistance-associated protein (MRP). Depending on ATP, the members of the MRP family can transport both hydrophobic uncharged molecules and water-soluble anionic compounds [66]. The latter includes the substrates conjugated with anions, such as glutathione, glucuronate or sulfate [67]. Considering our data, Tcas-ql MRP (GT63) might be an important transporter for the quinone precursors in all gland secretory cells, which also transports some alkene precursors in abdominal glands. And the transportations may occur from the hemolymph to glandular cells, or from the secreting cells to the vesicular compartments, where the toxicant-producing reactions are being segregated to [14].

Despite of these predictions on the functions of the three *quinone-less* gene encoded proteins based on their homologs, it should be noted that our phylogenic analyses show that they evolved independently and particularly for the chemical defense in the red flour beetle. Moreover, based on the GWMFISH results that confirm their expressions in the secretory units, all three genes play essential roles in producing the defensive quinones in the odoriferous glands of *T. castaneum*.

In chemical secretion of *Tribolium* and other quinone-producing arthropods, the quinones and the alkenes (hydrocarbons) have their respective roles. Quinones are toxic and quite reactive, therefore mainly responsible for the defense against pathogens, parasitoids, and predators [5]. Alkenes and mixtures of other organic solvents partition quinones to the hydrocarbon phase, help to spread them on the arthropod cuticle, and aid penetration of quinones through the cuticles of various enemies to improve the defensive effects [5], [68], [69]. Our inhibition assay results confirm these descriptions, since the *quinone-less* gene knock-downs (*Tcas-ql MRP*, *GT63*) showed no microbe inhibitions at all, whereas the *alkene-less* gene knock-down (*GT12*) presented only reduced inhibition effects.

In insects, innate immune responses include antimicrobial peptides, phagocytosis, nodulation, melanotic encapsulation and wound healing, which endow potential hosts the abilities to defend against pathogens and parasites [70]–[74]. The chemical defense system is also responsible for defending the host from infection by other organisms. Therefore, it was interesting to check how innate immunity was affected, when the chemical defense system is knocked-down. An oxidoreductase (PO) activity has been commonly assayed to provide a general index of melanization innate immune responses in invertebrates [46], since the activation of PO leads to the melanization reactions of invading pathogens, which is a major aspect of the innate immune system [75]. Therefore, PO activities were examined in *quinone-less* gene knock-downs compared to wild-type, buffer injected and EGFP dsRNA injected beetles. The obtained results indicate that the chemical defense may be linked with melanotic encapsulation innate immune responses. Thus the three *quinone-less* genes necessary for quinone biosynthesis in odoriferous glands might also be involved in the melanization cascade. In addition, the genomic annotation and expression analyses of the *Tribolium* POs showed that some POs are highly expressed in all gland samples but low in control, which implies that they might mainly function in the chemical defense system, and their preferred substrates are in the glands. However, whether they are involved in the melanin pathway is so far unknown. In case the POs are multifunctional, a linkage and crosstalk between these two systems would exist.

## Materials and Methods

### Transcriptome sequencing

Prothoracic and abdominal odoriferous glands were dissected separately from A10–A30 (reared at 32.5°C, 10–30 days after eclosion) adult beetles and stored in RNA*later* solution (Ambion, Life Technologies GmbH, Darmstadt, Germany, Cat. No. AM7020) on ice. Males and females were separately prepared except for the prothoracic glands from *tar* mutants. About 500 beetles were used for each gland sample, while the anterior abdomen, where no glands are located, was taken as a control tissue. Then total RNA was extracted using RNAqueous-Micro Kit (Ambion, Life Technologies GmbH, Darmstadt, Germany, Cat. No. AM1931) and treated by DNase I. Transcriptome sequencing (mRNA-seq) was performed by Macrogen Inc. (Seoul, South Korea), on a next generation sequencing (NGS) platform (Illumina/Solexa Genome Analyzer IIx). After sequencing, reads (38 bp each) were mapped to the mRNAs of the official gene set (OGS) from Beetlebase 3.0 [22], [38] by Maq tool (http://maq.sourceforge.net/). The samples (s) were, s1: anterior abdomen; s2: prothoracic glands from *tar* mutant; s3: male prothoracic glands; s4: female prothoracic glands; s5: male abdominal glands; s6: female abdominal glands. Except of s2, all other tissues were from wild-type. Coverage (depth) is calculated as reads times 38 divided by specific length of gene transcript. The transcriptomic sequences derived from the different samples have also been mapped as individual tracks to the *Tribolium* genome (iBeetle Genome Browser: http://bioinf.uni-greifswald.de/gb2/gbrowse/tcas).

### Gene ontology annotation and mRNA-seq library subtractions

The genes, which had coverage over 50 (about 2 times of the whole sequencing coverage), were regarded as abundant or richly expressed in either all wild-type gland samples or control. Their functionalities were explored by gene ontology (GO) annotation [39] using Blast2go [76], [77]. In order to screen gland-specific genes, statistical subtractions were carried out among different samples for various comparisons. In general, the cut-off for logarithm of fold change, with 2 as the base, was 6, which meant 64 times more reads in one sample than the other. The detailed subtraction conditions are presented in ***Dataset S2***.

### Transcriptomic exploration of candidate genes for quinone synthesis

*Tribolium* glucosidase, phenol oxidase and peroxidase were searched initially in protein database at the National Center for Biotechnology Information (NCBI) (http://www.ncbi.nlm.nih.gov/protein/). The obtained proteins were characterized based on conserved domains (CDD of NCBI, http://www.ncbi.nlm.nih.gov/Structure/cdd/cdd.shtml) and probed back to the publicly accessible *Tribolium* genome at Beetlebase with blastp algorithm in order to be linked with OGS, avoid redundancies, and identify the homologs which were not covered by the previous searches. The newly identified proteins were then analyzed in CDD for confirmation.

### Functional analysis of the most highly and gland-specifically expressed genes

To evaluate the subtraction results, 77 candidate genes were chosen from the gland transcriptome screening and functional analysis was performed by using RNA interference (RNAi) [78], [79]. An online tool, E-RNAi [80] was used to design fragments for double stranded RNA (dsRNA) synthesis with no or lowest off-target effects. Primers were designed by Primer Premier 5 [81] and listed in ***Dataset S5***. Animals were injected with dsRNAs at mid pupal [79] or larval L5–L6 stage [82], and were checked at A10 and A24 (32.5°C) for morphological phenotypes on prothoracic and abdominal glands. Furthermore, both pairs of glands were dissected carefully and intact from one male and one female beetle and smashed in 100 µl methanol (Merck Millipore SupraSolv, Merck KGaA, Darmstadt, Germany, Cat. No. 106011), which represents a more specific gland volatile analysis than previously performed by Howard 1987 (whole beetle [17]) or Villaverde *et al.* 2007 (headspace [9]). Then the samples were stored at −20°C and measured within 24 hours. One microliter was loaded by a split injector into an Agilent gas chromatograph coupled with a mass spectrometer (GC-MS) (Detailed parameters are described in ***Text S1***). The areas of the signals in chromatograms were calculated using the software MSD ChemStation D.02.00.275 (Agilent Technologies, Santa Clara, USA) under auto-integration mode. Then the data were compared between each candidate gene knock-down and the control. The phenotypes were grouped according to strengths of the alterations of the major components. For the three genes with quinone-less phenotypes, second independent dsRNA fragments, which had no overlaps with the first fragments, were designed with the same tools and used to confirm the phenotypes.

### Quantification of volatile gland contents

In order to quantify different volatile components in the secretion, the following chemicals were obtained from commercial sources: methyl-1,4-benzoquinone (MBQ) (abcr GmbH & Co. KG, Karlsruhe, Germany, Cat. No. AB208176), 2-methylhydroquinone (MHQ) (abcr GmbH & Co. KG, Karlsruhe, Germany, Cat. No. AB132029), ethyl quinol (also called ethyl-1,4-hydroquinone: EHQ) (abcr GmbH & Co. KG, Karlsruhe, Germany, Cat. No. AB148997), 1-pentadecene (Fluka, SIGMA-ALDRICH Chemie GmbH, Munich, Germany, Cat. No. 76560) and 1-heptadecene (Fluka, SIGMA-ALDRICH Chemie GmbH, Munich, Germany, Cat. No. 51665). Then authentic standard solution series were made and a five-point calibration was performed by GC-MS. Based on the standard curves, the areas of the abundances from GC-MS were transformed to masses. Ethyl-1,4-benzoquinone (EBQ) and heptadecadiene, which were commercially unavailable, were calculated as equivalents based on the standard curve of EHQ and 1-heptadecene respectively. Quantification was carried out in wild-type, buffer injected, a dsRNA EGFP injected control, and three *quinone-less* gene knock-downs. After pupal RNAi, glands were prepared from A10 beetles (15–30 animals each sex). It was proposed that more than 80% of the glandular quinones are benzoquinones [8]. Because the small amounts of hydroquinones detected are precursors of benzoquinones [24], [64], the quantities of hydroquinones and benzoquinones were summed up and treated as secreted quinones. After quantification, statistical analyses were performed with software JMP 9.0.2 (SAS Institute, 2010) using student t-test for sex comparisons and a nonparametric method (Mann–Whitney–Wilcoxon) for group comparisons.

### Phylogeny of the three novel quinone-less proteins

Full length cDNAs obtained from RACE reactions (see ***Text S1***) were analyzed by the online tool ORF Finder (Open Reading Frame finder, http://www.ncbi.nlm.nih.gov/gorf/orfig.cgi) and translated to proteins. The amino acid sequences were submitted to NCBI to find the homologs through blastp search in Reference Proteins Database and the first fifty sequences were chosen, in which the *Tribolium* homologs were blasted again in Beetlebase (http://beetlebase.org/) to check redundancies and find the corresponding OGS numbers (listed in ***Dataset S10***) in order to analyze their expressions at the glandular transcriptome level. Then all proteins (listed in ***Dataset S7, S8, and S9***) were aligned by using MAFFT [83] and analyzed with FastTree [84] using maximum likelihood methods to construct dendrograms, which were displayed, marked and computed based on the branching frequencies (cut-off was 60%) using MEGA5 [85].

### Gland whole mount fluorescent *in situ* hybridization

The protocol for gland whole mount fluorescent *in situ* hybridization (GWMFISH) was based on previous methods [86]–[90] with a few modifications. Details are described in ***Text S1***. The only big difference was using the HNPP Fluorescent Detection Set (Roche Applied Science, Roche Diagnostics Deutschland GmbH, Mannheim, Germany, Cat. No. 11758888001) to detect the labeled probes after incubation of Anti-digoxigenin (DIG)-alkaline phosphatase (AP) Fab fragments (Roche Applied Science, Roche Diagnostics Deutschland GmbH, Mannheim, Germany, Cat. No. 11093274910; from sheep). Glands were counterstained with Hoechst 33342 (SIGMA-ALDRICH Chemie GmbH, Munich, Germany, Cat. No. B2261) prior to mounting and embedding in Aqua-Poly/Mount (Polyscience, Niles, Illinois, USA, Cat. No. 18606). The stainings were observed and captured with confocal laser scanning microscope Zeiss LSM780. 3D (3-dimensional) construction was performed using software ZEN2011 (Carl Zeiss MicroImaging GmbH, Oberkochen, Germany). Contrast and brightness were adjusted using Adobe Photoshop CS2.

### Microbe inhibition assays

A fungus, *Aspergillus niger*, and a gram positive bacterium, *Arthrobacter globiformis* [91], were used to test the strength of the chemical defense. The *A. niger* strain was an isolate from old beetle cultures (GJ, unpublished), which was determined by the German collection of microorganisms and cell cultures (DSMZ) as *Aspergillus niger*, a common soil fungus also growing e.g. on bread and other food, known as ‘Black mold’. *A. globiformis* (from DSMZ, strain DSM20124) was another basic soil microbe and believed to have no contacts with *Tribolium* in nature. The culture media are listed in ***Text S1***. Microbe lawns were made in the petri dishes using the method of [44], [92], by the modification that we put dissected abdominal glands to the holes on the lawn poked by a sterile glass pipet (one pair of glands per hole and breaking of the reservoirs in the holes) instead of freezing beetles on the plates. The plates were incubated at 25°C for 72 h (*A. niger*) or 28°C for 48 h (*A. globiformis*) respectively, and then the inhibition zones were photographed with a digital camera. The areas of the inhibition zones were measured with freeware ImageJ 1.44p.

### Phenol oxidase activity assays

After RNAi, A10 beetles were harvested and frozen individually at −80°C in 150 µl Bis-Tris buffer (0.1 M, pH 7.5, sterile filtered. Bis-Tris: Fluka, SIGMA-ALDRICH Chemie GmbH, Munich, Germany, Cat. No. 14880) for at least 24 hours. To the frozen samples, a sterile steel ball (Ø 3 mm) was added each, and samples were homogenized using a GenoGrinder tissue homogenizer for 30 seconds at a speed of 1000 strokes per minute. After grinding, samples were placed on ice immediately before centrifuging three times at 6200 rpm 4°C (Eppendorf centrifuge 5810R) for five minutes to remove beetle debris. After each centrifugation step the supernatant was transferred to a new tube on ice before being centrifuged again. For measuring actual PO activity, a flat bottom 96well plate was prepared on ice with 50 µl sterile deionized water and 50 µl Bis-Tris buffer. In each well 20 µl of an individual sample extract was pipetted, or 20 µl Bis-Tris buffer when the well was serving as a blank. As PO activates the transfer of DOPA to Dopamine in insects [93], we added 50 µl L-DOPA (3,4-Dihydroxy-L-phenylalanine, SIGMA-ALDRICH Chemie GmbH, Munich, Germany, Cat. No. D9628; 4 mg/ml in Bis-Tris buffer, sterile filtered) into each well on ice. As the addition of substrate starts the reaction, plates needed to go to the Eon Microplate Spectrophotometer (Biotek Instruments, Inc., Bad Friedrichshall, Germany) immediately. Plates were read at 490 nm and 37°C with readings every two minutes for 90 minutes. After correcting the self-darkening of the substrate by subtracting the blanks, PO activity was estimated as Vmax of the linear phase of the reaction on every individual sample well (also compare with previous data [94]).

## Supporting Information

Dataset S1 Gland transcriptome library. s1: sample1, anterior abdomen; s2: sample2, prothoracic glands from *tar* mutant; s3: sample 3, male prothoracic glands; s4: sample 4, female prothoracic glands; s5: sample 5, male abdominal glands; s6: sample 6, female abdominal glands (Except s2, all the other tissues were from wild-type). Fold change (FC) is calculated as log2[reads in one sample/reads in another sample]. For abbreviations see Table 1.
(XLSX)

Dataset S2 Library subtraction procedures and results, including list of genes for GO.
(XLSX)

Dataset S3 Gene ontology results in details.
(XLSX)

Dataset S4 Annotations and gland transcriptomic expression levels of quinone synthesis-related genes, such as glucosidases (Glu), phenol oxidases (PO) and peroxidases (Per). For abbreviations see Table 1.
(XLSX)

Dataset S5 77 candidate genes, including primers and Tm for PCR to clone dsRNA fragments and RACE PCR, as well as their RNAi-induced phenotypes.
(XLSX)

Dataset S6 Quantification of main gland volatiles, including standard series, and statistical analyses of sex and group comparisons for wild-type, EGFP-injection control, and the three *quinone-less* gene knock-downs.
(XLSX)

Dataset S7 Sequences for phylogenetic analysis of GT39 (Tcas-qlVTGl) homologs.
(TXT)

Dataset S8 Sequences for phylogenetic analysis of GT62 (Tcas-qlARSB) homologs.
(TXT)

Dataset S9 Sequences for phylogenetic analysis of GT63 (Tcas-qlMRP) homologs.
(TXT)

Dataset S10 *Tribolium* homologs of *quinone-less* genes and their gland transcriptomic expression levels. For abbreviations see Table 1.
(XLSX)

Figure S1 Secretory cell morphology of odoriferous glands. Dissected DAPI-stained odoriferous glands. A, prothoracic glands. B, abdominal glands. The arrows indicate the vesicular organelles of cell type 2 that have been described previously in *Tribolium castaneum* [14] and another tenebrionid beetle, *Eleodes longicollis*, [13], [14]. Scale bars: 50 µm.
(TIF)

Figure S2 GC-MS Chromatograms of wild-type and *tar* mutant odoriferous glands. GC-MS was performed in order to check potential chemical alterations in gland volatiles of *tar* mutants that have more darkly pigmented prothoracic glands but unaffected abdominal glands [31]. A, prothoracic glands, B, abdominal glands. Chromatograms show volatile detection from wild-type (upper blue) and *tar* mutants (lower black). The prothoracic glands of *tar* mutants presented very low levels of alkenes, while the abdominal glands showed no significant difference to wild-type beetles. The peaks are: 1 and 2: methyl-1,4-benzoquinone; 3 and 4: ethyl-1,4-benzoquinone; 5: methyl-1,4-hydroquinone; 6: ethyl-1,4-hydroquinone; 7: 1,6-pentadecadiene; 8: 1-pentadecene; 9: 1,2-dimethoxy-4-n-propylbenzene; 10: 1-Hexadecene; 11: 1,8-heptadecadiene; 12: 1-Heptadecene. Double bond positions in 1,6-pentadecadiene and 1,8-heptadecadiene have not been confirmed, since these chemicals were not identified in the NIST database, but only assigned to similar peaks based on previous data [20], [21].
(TIF)

Figure S3 Phenol oxidase (PO) activity assays of wild-type and novel *quinone-less* gene RNAi knock-downs in males. The Y-axis indicates the square root of PO Vmax, red boxes are boxplots, green lines represent the mean value, the gray line represents the grand mean, while the X-axis presents wild-type, control injections, and different RNAi-knock-downs (N = 12–15, but the buffer-injected had only 4 beetles). Buffer: buffer-injection control; EGFP: dsEGFP-injection control; GT39: *Tcas-ql VTGl*; GT62: *Tcas-ql ARSB*; GT63: *Tcas-ql MRP*. The asterisks (*) marked the t-test results comparing to wild-type: ***, p<0.001; **, 0.001<p<0.01; *, 0.01<p<0.05. Buffer- and EGFP-injected controls were not significantly different from wild-type.
(TIF)

Text S1 In a separate file, we provide descriptions on beetle culture, gland cytology, RNA extraction and cDNA library construction, cloning of 77 candidate genes, RACE PCR, photo imaging and processing, gas chromatography and mass spectrometry (GC-MS), microbe culturing for inhibition assays, gland whole mount fluorescent *in situ* hybridization (GWMFISH), and related references.
(DOCX)

## References

[1] Chapman AD (2009) Numbers of living species in Australia and the world. Second. Canberra, Australia: Australian Biological Resources Study (ABRS). 80 pp. Available:http://www.environment.gov.au/biodiversity/abrs/publications/other/species-numbers/2009/06-references.html. Accessed 9 August 2012.

[2] Wilson EO (2006) Threats to Global Diversity. Available:http://www.globalchange.umich.edu/globalchange2/current/lectures/biodiversity/biodiversity.html. Accessed 9 August 2012.

[3] Eisner T (1970) Chemical defense against predation in arthropods. In: Sondheimer E, Simeone JB, editors. Chemical Ecology. New York: Academic Press, Vol. Academic P. pp. 157–217.

[4] Eisner T (1966) Beetle's spray discourages predators. Natural History 75: 42–47.

[5] Blum MS (1981) Chemical defenses of arthropods. United Kin. London: Academic Press. 562 pp. Available:http://www.cabdirect.org/abstracts/19820594729.htmljsessionid=6D34AB6A23BB5FBEB2313F77658399B7. Accessed 9 August 2012.

[6] Eisner T, Meinwald J (1966) Defensive secretions of arthropods. Science 153: 1341–1350 Available:http://www.sciencemag.org/content/153/3742/1341.short. Accessed 9 June 2012. 17814381 10.1126/science.153.3742.1341

[7] Weatherston J (1967) The chemistry of arthropod defensive substances. Quarterly Reviews, Chemical Society 21: 287 Available:http://pubs.rsc.org/en/content/articlehtml/1967/qr/qr9672100287. Accessed 9 August 2012.

[8] Unruh LM, Xu R, Kramer KJ (1998) Benzoquinone levels as a function of age and gender of the red flour beetle, Tribolium castaneum. Insect Biochemistry and Molecular Biology 28: 969–977 Available:http://linkinghub.elsevier.com/retrieve/pii/S096517489800085X. Accessed 19 March 2012.

[9] Villaverde ML, Juárez MP, Mijailovsky S (2007) Detection of Tribolium castaneum (Herbst) volatile defensive secretions by solid phase microextraction–capillary gas chromatography (SPME-CGC). Journal of Stored Products Research 43: 540–545 Available:http://dx.doi.org/10.1016/j.jspr.2007.03.003. Accessed 3 April 2012.

[10] Chittenden FH (1896) Insects Affecting Cereals and Other Dry Vegetable Foods. Bulletin of the United States Department of Agriculture; Division of Entomology (New Series) 4: 112–130.

[11] Payne NM (1925) Some effects of Tribolium on flour. Journal of Economic Entomology 18: 737–744 Available:http://www.ingentaconnect.com/content/esa/jee/1925/00000018/00000005/art00020.

[12] Roth LM (1943) Studies on the gaseous secretion of Tribolium confusum Duval. II. The odoriferous glands of Tribolium confusum. Annals of the Entomological Society of America 36: 397–424 Available:http://www.ingentaconnect.com/content/esa/aesa/1943/00000036/00000003/art00011. Accessed 6 June 2012.

[13] Eisner T, McHenry F, Salpeter MM (1964) Defense mechanisms of arthropods. XV. Morphology of the quinone-producing glands of a tenebrionid beetle (ELEODES longicollis lec.). Journal of morphology 115: 355–399 Available:http://onlinelibrary.wiley.com/doi/10.1002/jmor.1051150304/abstract. Accessed 4 June 2012. 14234866 10.1002/jmor.1051150304

[14] Happ GM (1968) Quinone and hydrocarbon production in the defensive glands of Eleodes longicollis and Tribolium castaneum (Coleoptera, Tenebrionidae). Journal of insect physiology 14: 1821–1837 Available:http://www.sciencedirect.com/science/article/pii/002219106890214X.

[15] Alexander P, Barton DHR (1943) The excretion of ethylquinone by the flour beetle. The Biochemical Journal 37: 463–465 Available:http://www.pubmedcentral.nih.gov/articlerender.fcgi?artid=1257939&tool=pmcentrez&rendertype=abstract. Accessed 26 March 2012. 16747670 10.1042/bj0370463 PMC1257939

[16] Loconti JD, Roth LM (1953) Composition of the odorous secretion of Tribolium castaneum. Annals of the Entomological Society of America 46: 281–289 Available:http://www.ingentaconnect.com/content/esa/aesa/1953/00000046/00000002/art00011. Accessed 10 August 2012.

[17] Howard RW (1987) Chemosystematic studies of the Triboliini (Coleoptera: Tenebrionidae): phylogenetic inferences from the defensive chemicals of eight Tribolium spp., Palorus ratzeburgi (Wissmann), and latheticus oryzae Waterhouse. Annals of the Entomological Society of America 80: 398–405 Available:http://agris.fao.org/agris-search/search/display.do?f=1987/US/US87078.xmlUS8738541. Accessed 16 August 2012.

[18] Markarian H, Florentine GJ, Prait JJ Jr (1978) Quinone production of some species of Tribolium. Journal of Insect Physiology 24: 785–790 Available:http://www.sciencedirect.com/science/article/pii/0022191078900963. Accessed 10 August 2012.

[19] Von Endt DW, Wheeler JW (1971) 1-Pentadecene production in Tribolium confusum. Science 172: 60–61 Available:http://www.sciencemag.org/content/172/3978/60.short. Accessed 19 March 2012. 17736498 10.1126/science.172.3978.60

[20] Suzuki T, Huynh V, Muto T (1975) Hydrocarbon repellents isolated from Tribolium castaneum and T. confusum (Coleoptera: Tenebrionidae). Agricultural and Biological Chemistry 39: 2207–2211 Available:http://agris.fao.org/agris-search/search/display.do?f=1976/JP/JP76004.xmlJP7601784.

[21] Görgen G, Frößl C, Boland W, Dettner K (1990) Biosynthesis of 1-alkenes in the defensive secretions of Tribolium confusum (Tenebrionidae); stereochemical implications. Experientia 46: 700–704 Available:http://www.springerlink.com/content/hx288200k15331hr/. Accessed 10 August 2012.

[22] Wang L, Wang S, Li Y, Paradesi MSR, Brown SJ (2007) BeetleBase: the model organism database for Tribolium castaneum. Nucleic acids research 35: D476–9 Available:http://www.pubmedcentral.nih.gov/articlerender.fcgi?artid=1669707&tool=pmcentrez&rendertype=abstract. Accessed 9 May 2012. 17090595 10.1093/nar/gkl776 PMC1669707

[23] Bucher G, Scholten J, Klingler M (2002) Parental RNAi in Tribolium (Coleoptera). Current biology: CB 12: R85–6 Available:http://www.ncbi.nlm.nih.gov/pubmed/11839285. Accessed 13 August 2012. 11839285 10.1016/s0960-9822(02)00666-8

[24] Tomoyasu Y, Miller SC, Tomita S, Schoppmeier M, Grossmann D, et al (2008) Exploring systemic RNA interference in insects: a genome-wide survey for RNAi genes in Tribolium. Genome Biology 9: R10 Available:http://www.pubmedcentral.nih.gov/articlerender.fcgi?artid=2395250&tool=pmcentrez&rendertype=abstract. 18201385 10.1186/gb-2008-9-1-r10 PMC2395250

[25] Lorenzen MD, Doyungan Z, Savard J, Snow K, Crumly LR, et al (2005) Genetic linkage maps of the red flour beetle, Tribolium castaneum, based on bacterial artificial chromosomes and expressed sequence tags. Genetics 170: 741–747 Available:http://www.pubmedcentral.nih.gov/articlerender.fcgi?artid=1450394&tool=pmcentrez&rendertype=abstract. Accessed 8 March 2012. 15834150 10.1534/genetics.104.032227 PMC1450394

[26] Trauner J, Schinko J, Lorenzen MD, Shippy TD, Wimmer EA, et al (2009) Large-scale insertional mutagenesis of a coleopteran stored grain pest, the red flour beetle Tribolium castaneum, identifies embryonic lethal mutations and enhancer traps. BMC biology 7: 73 Available:http://www.biomedcentral.com/1741-7007/7/73. Accessed 5 March 2012. 19891766 10.1186/1741-7007-7-73 PMC2779179

[27] Schinko JB, Weber M, Viktorinova I, Kiupakis A, Averof M, et al (2010) Functionality of the GAL4/UAS system in Tribolium requires the use of endogenous core promoters. BMC developmental biology 10: 53 Available:http://www.pubmedcentral.nih.gov/articlerender.fcgi?artid=2882914&tool=pmcentrez&rendertype=abstract. Accessed 27 October 2012. 20482875 10.1186/1471-213X-10-53 PMC2882914

[28] Schinko JB, Hillebrand K, Bucher G (2012) Heat shock-mediated misexpression of genes in the beetle Tribolium castaneum. Development genes and evolution 222: 287–298 Available:http://www.ncbi.nlm.nih.gov/pubmed/22890852. Accessed 27 October 2012. 22890852 10.1007/s00427-012-0412-x

[29] Tribolium Genome Sequencing Consortium (2008) Richards S, Gibbs RA, Weinstock GM, Brown SJ, et al (2008) The genome of the model beetle and pest Tribolium castaneum. Nature 452: 949–955 Available:http://dx.doi.org/10.1038/nature06784. Accessed 1 March 2012. 18362917 10.1038/nature06784

[30] Engelhardt M, Rapoport H, Sokoloff A (1965) Odorous secretion of normal and mutant Tribolium confusum. Science (New York, NY) 150: 632–633 Available:http://www.ncbi.nlm.nih.gov/pubmed/5837106. Accessed 13 August 2012. 10.1126/science.150.3696.632 5837106

[31] Beeman RW, Stuart JJ, Haas MS, Friesen KS (1992) Chromosome extraction and revision of linkage group 2 in Tribolium castaneum. The Journal of heredity 87: 224–232 Available:http://www.ncbi.nlm.nih.gov/pubmed/8683098. 10.1093/oxfordjournals.jhered.a022989 8683098

[32] Meinwald J, Koch KF, Rogers JE, Eisner T (1966) Biosynthesis of Arthropod Secretions. III. Synthesis of Simple p-Benzoquinones in a Beetle (Eleodes longicollis). Journal of the American chemical society 88: 1590–1592 Available:http://dx.doi.org/10.1021/ja00959a074. Accessed 9 June 2012.

[33] Morgan ED (2004) Biosynthesis in Insects. Cambridge: Royal Society of Chemistry. 199 pp. Available:http://pubs.rsc.org/en/content/ebook/978-0-85404-691-1.

[34] Duffey SS, Blum MS (1977) Phenol and guaiacol: Biosynthesis, detoxication, and function in a polydesmid millipede, Oxidus gracilis. Insect Biochemistry 7: 57–65.

[35] Pryor MGM (1940) On the hardening of the cuticle of insects. Proc R Soc London Ser B128: 393–407.

[36] Kramer KJ, Kanost MR, Hopkins TL, Jiang H, Zhu YC, et al (2001) Oxidative conjugation of catechols with proteins in insect skeletal systems. Tetrahedron 57: 385–392 Available:http://dx.doi.org/10.1016/S0040-4020(00)00949-2. Accessed 12 March 2013.

[37] Suderman RJ, Dittmer NT, Kanost MR, Kramer KJ (2006) Model reactions for insect cuticle sclerotization: cross-linking of recombinant cuticular proteins upon their laccase-catalyzed oxidative conjugation with catechols. Insect biochemistry and molecular biology 36: 353–365 Available:http://www.ncbi.nlm.nih.gov/pubmed/16551549. Accessed 12 March 2013. 16551549 10.1016/j.ibmb.2006.01.012

[38] Kim HS, Murphy T, Xia J, Caragea D, Park Y, et al (2010) BeetleBase in 2010: revisions to provide comprehensive genomic information for Tribolium castaneum. Nucleic acids research 38: D437–42 Available:http://nar.oxfordjournals.org/cgi/content/abstract/38/suppl_1/D437. Accessed 6 June 2012. 19820115 10.1093/nar/gkp807 PMC2808946

[39] The Gene Ontology Consortium (2000) Ashburner M, Ball CA, Blake JA, Botstein D, et al (2000) Gene Ontology: tool for the unification of biology. Nature genetics 25: 25–29 Available:http://www.pubmedcentral.nih.gov/articlerender.fcgi?artid=3037419&tool=pmcentrez&rendertype=abstract. Accessed 8 March 2012. 10802651 10.1038/75556 PMC3037419

[40] Ladish RK, Ladish SK, Howe PM (1967) Quinoid Secretions in Grain and Flour Beetles. Nature 215: 939–940 Available:http://dx.doi.org/10.1038/215939a0. Accessed 16 August 2012. 6055420 10.1038/215939a0

[41] Wirtz RA, Taylor SL, Semey HG (1978) Concentrations of substituted p-benzoquinones and 1-pentadecene in the flour beetles Tribolium madens (Charp.) and Tribolium Brevicornis (Lec.) (Coleoptera, Tenebrionidae). Comparative biochemistry and physiology 61: 287–290 Available:http://www.sciencedirect.com/science/article/pii/0306449278900564.318376

[42] Pappas PW, Wardrop SM (1996) Quantification of benzoquinones in the flour beetles, Tribolium castaneum and Tribolium confusum. Preparative biochemistry & biotechnology 26: 53–66 Available:http://www.ncbi.nlm.nih.gov/pubmed/8744422. Accessed 26 March 2012. 8744422 10.1080/10826069608000050

[43] Yezerski A, Gilmor TP, Stevens L (2004) Genetic analysis of benzoquinone production in Tribolium confusum. Journal of chemical ecology 30: 1035–1044 Available:http://www.ncbi.nlm.nih.gov/pubmed/15274446. 15274446 10.1023/b:joec.0000028465.37658.ae

[44] Prendeville HR, Stevens L (2002) Microbe inhibition by Tribolium flour beetles varies with beetle species, strain, sex, and microbe group. Journal of chemical ecology 28: 1183–1190 Available:http://www.ncbi.nlm.nih.gov/pubmed/12184396. 12184396 10.1023/a:1016281600915

[45] Yezerski A, Ciccone C, Rozitski J, Volingavage B (2007) The effects of a naturally produced benzoquinone on microbes common to flour. Journal of chemical ecology 33: 1217–1225 Available:http://www.ncbi.nlm.nih.gov/pubmed/17473960. Accessed 26 March 2012. 17473960 10.1007/s10886-007-9293-2

[46] Armitage S, Siva-Jothy M (2005) Immune function responds to selection for cuticular colour in Tenebrio molitor. Heredity 94: 650–656 Available:http://www.ncbi.nlm.nih.gov/pubmed/15815710. Accessed 20 March 2012. 15815710 10.1038/sj.hdy.6800675

[47] Sokoloff A (1966) The genetics of Tribolium and related species. Advances in genetics, Supplement 1. New York: Academic Press. 212 pp.

[48] Leal WS (2005) Pheromone reception. Topics in Current Chemistry 240: 1–36 Available:http://www.springerlink.com/index/WV1PY7HYC4861T2D.pdf. Accessed 22 August 2012.

[49] Pelletier J, Leal WS (2009) Genome analysis and expression patterns of odorant-binding proteins from the Southern House mosquito Culex pipiens quinquefasciatus. PloS one 4: e6237 Available:http://dx.plos.org/10.1371/journal.pone.0006237. Accessed 22 August 2012. 19606229 10.1371/journal.pone.0006237 PMC2707629

[50] Roth LM, Eisner T (1962) Chemical defenses of arthropods. Annual Review of Entomology 7: 107–136 Available:http://www.annualreviews.org/doi/abs/10.1146/annurev.en.07.010162.000543? Accessed 9 August 2012.

[51] Sokoloff A (1974) The Biology of Tribolium. Vol. 2. Oxford: Clarendon Press. 628 pp.

[52] Cavill GWK (1971) Chemistry of Some Insect Secretions. Journal and Proceedings of the Royal Society of New South Wales 103: 109–118 Available:http://www.ncbi.nlm.nih.gov/pubmed/10440781.

[53] Fowler K, Partridge L (1989) A cost of mating in female fruitflies. Nature 338: 760–761 Available:http://dx.doi.org/10.1038/338760a0. Accessed 11 November 2012.

[54] Chapman T, Liddle LF, Kalb JM, Wolfner MF, Partridge L (1995) Cost of mating in Drosophila melanogaster females is mediated by male accessory gland products. Nature 373: 241–244 Available:http://dx.doi.org/10.1038/373241a0. Accessed 6 November 2012. 7816137 10.1038/373241a0

[55] Kemp DJ, Rutowski RL (2004) A survival cost to mating in a polyandrous butterfly, Colias eurytheme. Oikos 105: 65–70 Available:http://doi.wiley.com/10.1111/j.0030-1299.2004.12874.x. Accessed 11 November 2012.

[56] Gilg MR, Kruse KC (2003) Reproduction Decreases Life Span in the Giant Waterbug (Belostoma flumineum). The American Midland Naturalist 149: 306–319 Available:http://dx.doi.org/10.1674/0003-0031(2003)149[0306:RDLSIT]2.0.CO;2. Accessed 11 November 2012.

[57] Rönn J, Katvala M, Arnqvist G (2006) The costs of mating and egg production in Callosobruchus seed beetles. Animal Behaviour 72: 335–342 Available:http://dx.doi.org/10.1016/j.anbehav.2005.10.024. Accessed 11 November 2012.

[58] Tufail M, Takeda M (2009) Insect vitellogenin/lipophorin receptors: molecular structures, role in oogenesis, and regulatory mechanisms. Journal of insect physiology 55: 87–103 Available:http://www.ncbi.nlm.nih.gov/pubmed/19071131. Accessed 14 October 2012. 19071131 10.1016/j.jinsphys.2008.11.007

[59] Lee KM, Lee KY, Choi HW, Cho MY, Kwon TH, et al (2000) Activated phenoloxidase from Tenebrio molitor larvae enhances the synthesis of melanin by using a vitellogenin-like protein in the presence of dopamine. European Journal of Biochemistry 267: 3695–3703 Available:http://doi.wiley.com/10.1046/j.1432-1327.2000.01402.x. Accessed 28 May 2012. 10848987 10.1046/j.1432-1327.2000.01402.x

[60] Hearing VJ (2011) Determination of melanin synthetic pathways. The Journal of investigative dermatology 131: E8–E11 Available:http://www.ncbi.nlm.nih.gov/pubmed/22094404. Accessed 30 October 2012. 22094404 10.1038/skinbio.2011.4 PMC6944209

[61] Eisenman HC, Casadevall A (2012) Synthesis and assembly of fungal melanin. Applied microbiology and biotechnology 93: 931–940 Available:http://www.ncbi.nlm.nih.gov/pubmed/22173481. Accessed 30 October 2012. 22173481 10.1007/s00253-011-3777-2 PMC4318813

[62] U.S. National Library of Medicine (2010) ARSB. Genetics Home Reference Available:http://ghr.nlm.nih.gov/gene/ARSB.

[63] Litjens T, Hopwood JJ (2001) Mucopolysaccharidosis type VI: Structural and clinical implications of mutations in N-acetylgalactosamine-4-sulfatase. Human mutation 18: 282–295 Available:http://www.ncbi.nlm.nih.gov/pubmed/11668612. Accessed 26 August 2012. 11668612 10.1002/humu.1190

[64] Neufeld E, Muenzer J (2001) The mucopolysaccharidoses. In: Scriver C, Beaudet A, Sly W, Valle D, editors. The metabolic and molecular bases of inherited disease. New York: McGraw-Hill. pp. 3421–3452.

[65] Saftig P, Klumperman J (2009) Lysosome biogenesis and lysosomal membrane proteins: trafficking meets function. Nature reviews Molecular cell biology 10: 623–635 Available:http://www.ncbi.nlm.nih.gov/pubmed/19672277. Accessed 26 October 2012. 19672277 10.1038/nrm2745

[66] Glavinas H, Krajcsi P, Cserepes J, Sarkadi B (2004) The role of ABC transporters in drug resistance, metabolism and toxicity. Current drug delivery 1: 27–42 Available:http://www.ncbi.nlm.nih.gov/pubmed/16305368. Accessed 27 August 2012. 16305368 10.2174/1567201043480036

[67] Homolya L, Váradi A, Sarkadi B (2003) Multidrug resistance-associated proteins: Export pumps for conjugates with glutathione, glucuronate or sulfate. BioFactors (Oxford, England) 17: 103–114 Available:http://www.ncbi.nlm.nih.gov/pubmed/12897433. Accessed 23 August 2012. 10.1002/biof.5520170111 12897433

[68] Peschke K, Eisner T (1987) Defensive secretion of the tenebrionid beetle,Blaps mucronata: Physical and chemical determinants of effectiveness. Journal of Comparative Physiology A 161: 377–388 Available:http://www.springerlink.com/content/x06t6p8200072x86/. Accessed 9 June 2012. 10.1007/BF00603963 3668879

[69] Dettner K (1991) Solvent-dependent variability of effectiveness of quinone-defensive systems of Oxytelinae beetles (Coleoptera: Staphylinidae). Entomologia Generalis 15: 275–292.

[70] Ashida M, Brey P (1998) Recent advances on the research of the insect prophenoloxidase cascade. In: Brey P, Hultmark D, editors. Molecular mechanisms of immune responses in insects. London, UK: Chapman & Hall. pp. 135–172.

[71] Cerenius L, Söderhäll K (2004) The prophenoloxidase-activating system in invertebrates. Immunological reviews 198: 116–126 Available:http://www.ncbi.nlm.nih.gov/pubmed/15199959. 15199959 10.1111/j.0105-2896.2004.00116.x

[72] Kanost MR, Jiang H, Yu X-Q (2004) Innate immune responses of a lepidopteran insect, Manduca sexta. Immunological reviews 198: 97–105 Available:http://www.ncbi.nlm.nih.gov/pubmed/15199957. Accessed 31 July 2012. 15199957 10.1111/j.0105-2896.2004.0121.x

[73] Mavrouli MD, Tsakas S, Theodorou GL, Lampropoulou M, Marmaras VJ (2005) MAP kinases mediate phagocytosis and melanization via prophenoloxidase activation in medfly hemocytes. Biochimica et biophysica acta 1744: 145–156 Available:http://www.ncbi.nlm.nih.gov/pubmed/15921769. Accessed 31 July 2012. 15921769 10.1016/j.bbamcr.2005.04.011

[74] Vilcinskas A (2013) Evolutionary plasticity of insect immunity. Journal of insect physiology 59: 123–129 Available:http://www.ncbi.nlm.nih.gov/pubmed/22985862. Accessed 11 March 2013. 22985862 10.1016/j.jinsphys.2012.08.018

[75] Söderhäll K, Cerenius L (1998) Role of the prophenoloxidase-activating system in invertebrate immunity. Current Opinion in Immunology 10: 23–28 Available:http://dx.doi.org/10.1016/S0952-7915(98)80026-5. Accessed 5 July 2012. 9523106 10.1016/s0952-7915(98)80026-5

[76] Conesa A, Götz S, García-Gómez JM, Terol J, Talón M, et al (2005) Blast2GO: a universal tool for annotation, visualization and analysis in functional genomics research. Bioinformatics 21: 3674–3676 Available:http://bioinformatics.oxfordjournals.org/cgi/content/abstract/21/18/3674. Accessed 9 March 2012. 16081474 10.1093/bioinformatics/bti610

[77] Götz S, García-Gómez JM, Terol J, Williams TD, Nagaraj SH, et al (2008) High-throughput functional annotation and data mining with the Blast2GO suite. Nucleic acids research 36: 3420–3435 Available:http://nar.oxfordjournals.org/cgi/content/abstract/36/10/3420. Accessed 3 March 2012. 18445632 10.1093/nar/gkn176 PMC2425479

[78] Hannon G (2002) RNA interference. Nature 418: 24–26 Available:http://codex.cshl.org/publications/Hannon_2002_12110901.pdf. Accessed 1 June 2012.

[79] Posnien N, Schinko J, Grossmann D, Shippy TD, Konopova B, et al (2009) RNAi in the red flour beetle (Tribolium). Cold Spring Harbor protocols 2009: pdb.prot5256 Available:http://www.ncbi.nlm.nih.gov/pubmed/20147232. Accessed 9 May 2012. 20147232 10.1101/pdb.prot5256

[80] Horn T, Boutros M (2010) E-RNAi: a web application for the multi-species design of RNAi reagents–2010 update. Nucleic acids research 38: W332–9 Available:http://nar.oxfordjournals.org/cgi/content/abstract/38/suppl_2/W332. Accessed 12 March 2012. 20444868 10.1093/nar/gkq317 PMC2896145

[81] Lalitha S (2000) Primer Premier 5. Biotech Software & Internet Report 1: 270–272 Available:http://online.liebertpub.com/doi/abs/10.1089%2F152791600459894.

[82] Tomoyasu Y, Denell RE (2004) Larval RNAi in Tribolium (Coleoptera) for analyzing adult development. Development genes and evolution 214: 575–578 Available:http://www.ncbi.nlm.nih.gov/pubmed/15365833. Accessed 2 April 2012. 15365833 10.1007/s00427-004-0434-0

[83] Katoh K, Kuma K, Toh H, Miyata T (2005) MAFFT version 5: improvement in accuracy of multiple sequence alignment. Nucleic acids research 33: 511–518 Available:http://nar.oxfordjournals.org/cgi/content/abstract/33/2/511. Accessed 11 March 2012. 15661851 10.1093/nar/gki198 PMC548345

[84] Price MN, Dehal PS, Arkin AP (2010) FastTree 2–approximately maximum-likelihood trees for large alignments. PloS one 5: e9490 Available:http://dx.plos.org/10.1371/journal.pone.0009490. Accessed 12 March 2012. 20224823 10.1371/journal.pone.0009490 PMC2835736

[85] Tamura K, Peterson D, Peterson N, Stecher G, Nei M, et al (2011) MEGA5: molecular evolutionary genetics analysis using maximum likelihood, evolutionary distance, and maximum parsimony methods. Molecular biology and evolution 28: 2731–2739 Available:http://www.pubmedcentral.nih.gov/articlerender.fcgi?artid=3203626&tool=pmcentrez&rendertype=abstract. Accessed 29 February 2012. 21546353 10.1093/molbev/msr121 PMC3203626

[86] Friedrich M, Benzer S (2000) Divergent decapentaplegic expression patterns in compound eye development and the evolution of insect metamorphosis. The Journal of experimental zoology 288: 39–55 Available:http://www.ncbi.nlm.nih.gov/pubmed/10750052. Accessed 14 May 2012. 10750052 10.1002/(sici)1097-010x(20000415)288:1<39::aid-jez5>3.0.co;2-t

[87] Osborne P, Dearden PK (2005) Non-radioactive in-situ hybridisation to honeybee embryos and ovaries. Apidologie 36: 113–118 doi:10.1051/apido

[88] Schinko J, Posnien N, Kittelmann S, Koniszewski N, Bucher G (2009) Single and double whole-mount in situ hybridization in red flour beetle (Tribolium) embryos. Cold Spring Harbor protocols 2009: pdb.prot5258 Available:http://cshprotocols.cshlp.org/cgi/content/abstract/2009/8/pdb.prot5258. Accessed 8 June 2012. 20147234 10.1101/pdb.prot5258

[89] Suzuki Y, Squires DC, Riddiford LM (2009) Larval leg integrity is maintained by Distal-less and is required for proper timing of metamorphosis in the flour beetle, Tribolium castaneum. Developmental biology 326: 60–67 Available:http://www.pubmedcentral.nih.gov/articlerender.fcgi?artid=2762819&tool=pmcentrez&rendertype=abstract. Accessed 7 June 2012. 19022238 10.1016/j.ydbio.2008.10.022 PMC2762819

[90] Asp J, Abramsson A, Betsholtz C (2006) Nonradioactive in situ hybridization on frozen sections and whole mounts. Methods in molecular biology (Clifton, NJ) 326: 89–102 Available:http://www.ncbi.nlm.nih.gov/pubmed/16780195. 10.1385/1-59745-007-3:89 16780195

[91] Conn HJ, Dimmick I (1947) Soil Bacteria Similar in Morphology to Mycobacterium and Corynebacterium. Journal of bacteriology 54: 291–303 Available:http://www.pubmedcentral.nih.gov/articlerender.fcgi?artid=526554&tool=pmcentrez&rendertype=abstract. Accessed 7 June 2012. 16561362 10.1128/jb.54.3.291-303.1947 PMC526554

[92] Faye I, Wyatt GR (1980) The synthesis of antibacterial proteins in isolated fat body from Cecropia silkmoth pupae. Experientia 36: 1325–1326 Available:http://www.ncbi.nlm.nih.gov/pubmed/7449923. Accessed 28 October 2012. 7449923 10.1007/BF01969615

[93] Cerenius L, Lee BL, Söderhäll K (2008) The proPO-system: pros and cons for its role in invertebrate immunity. Trends in immunology 29: 263–271 Available:http://www.ncbi.nlm.nih.gov/pubmed/18457993. Accessed 17 July 2012. 18457993 10.1016/j.it.2008.02.009

[94] Roth O, Joop G, Eggert H, Hilbert J, Daniel J, et al (2010) Paternally derived immune priming for offspring in the red flour beetle, Tribolium castaneum. The Journal of animal ecology 79: 403–413 Available:http://www.ncbi.nlm.nih.gov/pubmed/19840170. Accessed 30 March 2012. 19840170 10.1111/j.1365-2656.2009.01617.x

