# Supplementary figures and images for: Odoriferous Defensive Stink Gland Transcriptome to Identify Novel Genes Necessary for Quinone Synthesis in the Red Flour Beetle, Tribolium castaneum
Source: PLoS Genet. 2013 Jul 11;9(7):e1003596. doi: 10.1371/journal.pgen.1003596 (PMC3708791; doi:10.1371/journal.pgen.1003596)

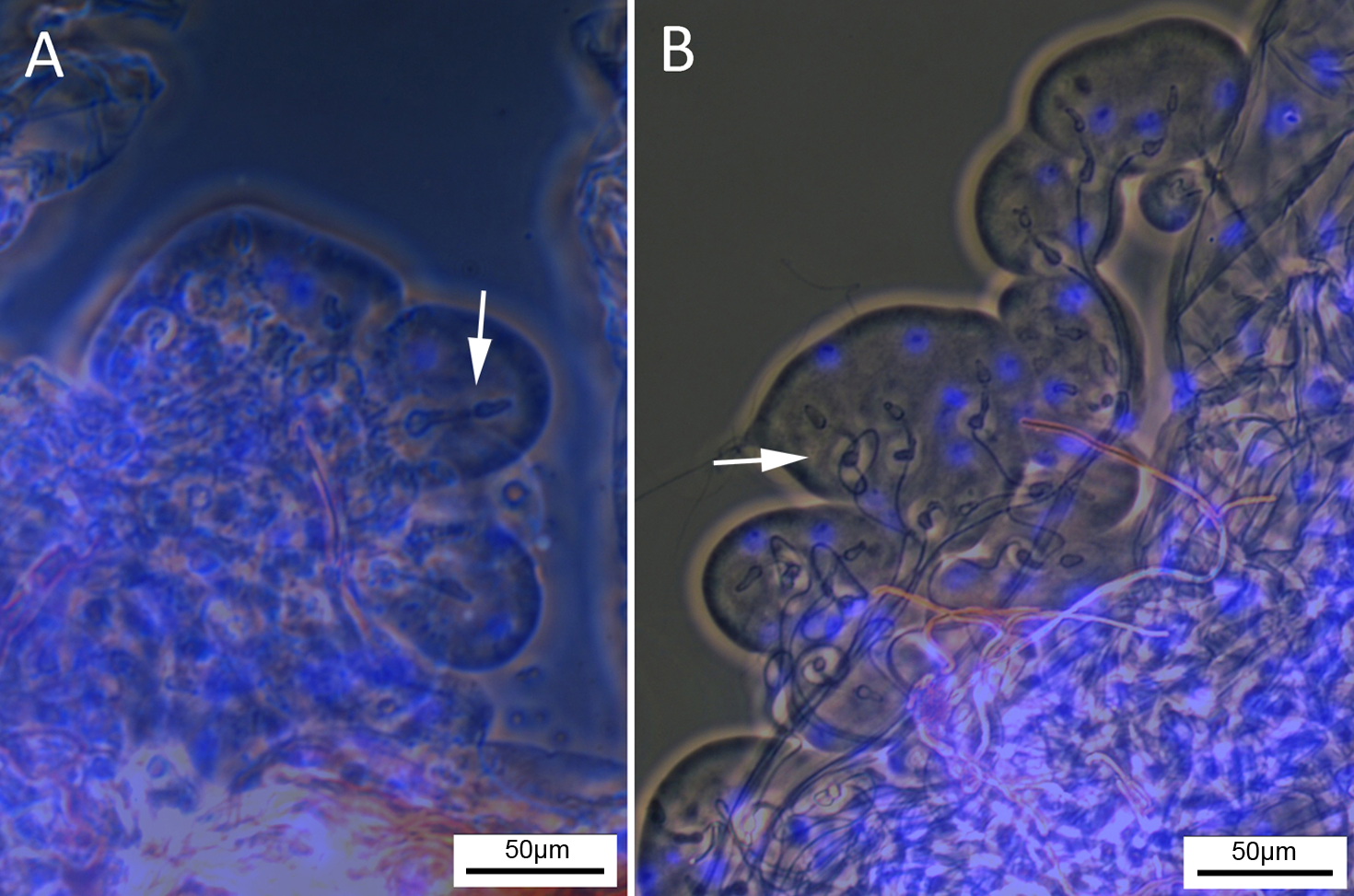

Supplement: Figure S1 — Secretory cell morphology of odoriferous glands. Dissected DAPI-stained odoriferous glands. A, prothoracic glands. B, abdominal glands. The arrows indicate the vesicular organelles of cell type 2 that have been described previously in Tribolium castaneum [14] and another tenebrionid beetle, Eleodes longicollis, [13], [14]. Scale bars: 50 µm. (TIF) [file pgen.1003596.s011.tif]

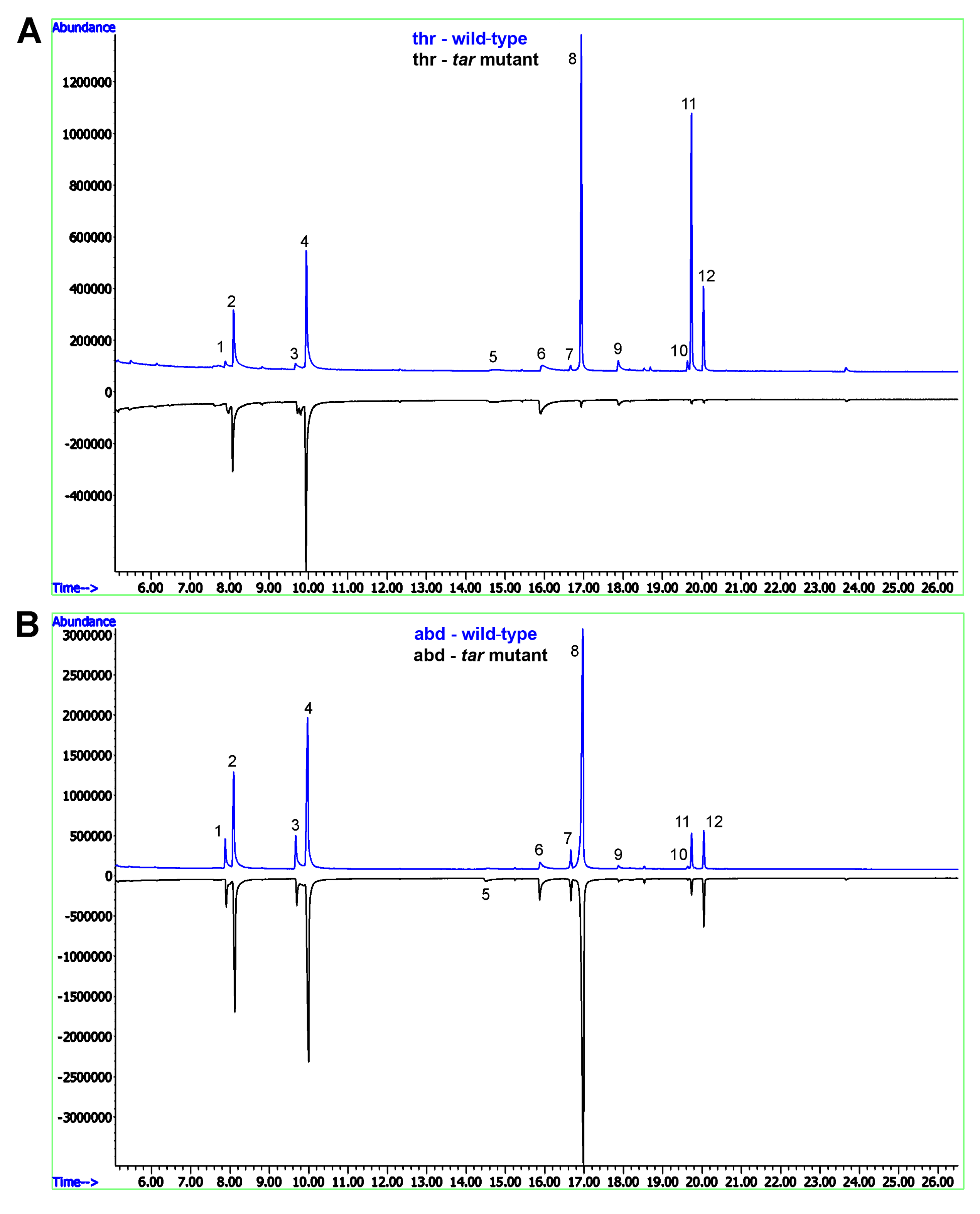

Supplement: Figure S2 — GC-MS Chromatograms of wild-type and tar mutant odoriferous glands. GC-MS was performed in order to check potential chemical alterations in gland volatiles of tar mutants that have more darkly pigmented prothoracic glands but unaffected abdominal glands [31]. A, prothoracic glands, B, abdominal glands. Chromatograms show volatile detection from wild-type (upper blue) and tar mutants (lower black). The prothoracic glands of tar mutants presented very low levels of alkenes, while the abdominal glands showed no significant difference to wild-type beetles. The peaks are: 1 and 2: methyl-1,4-benzoquinone; 3 and 4: ethyl-1,4-benzoquinone; 5: methyl-1,4-hydroquinone; 6: ethyl-1,4-hydroquinone; 7: 1,6-pentadecadiene; 8: 1-pentadecene; 9: 1,2-dimethoxy-4-n-propylbenzene; 10: 1-Hexadecene; 11: 1,8-heptadecadiene; 12: 1-Heptadecene. Double bond positions in 1,6-pentadecadiene and 1,8-heptadecadiene have not been confirmed, since these chemicals were not identified in the NIST database, but only assigned to similar peaks based on previous data [20], [21]. (TIF) [file pgen.1003596.s012.tif]

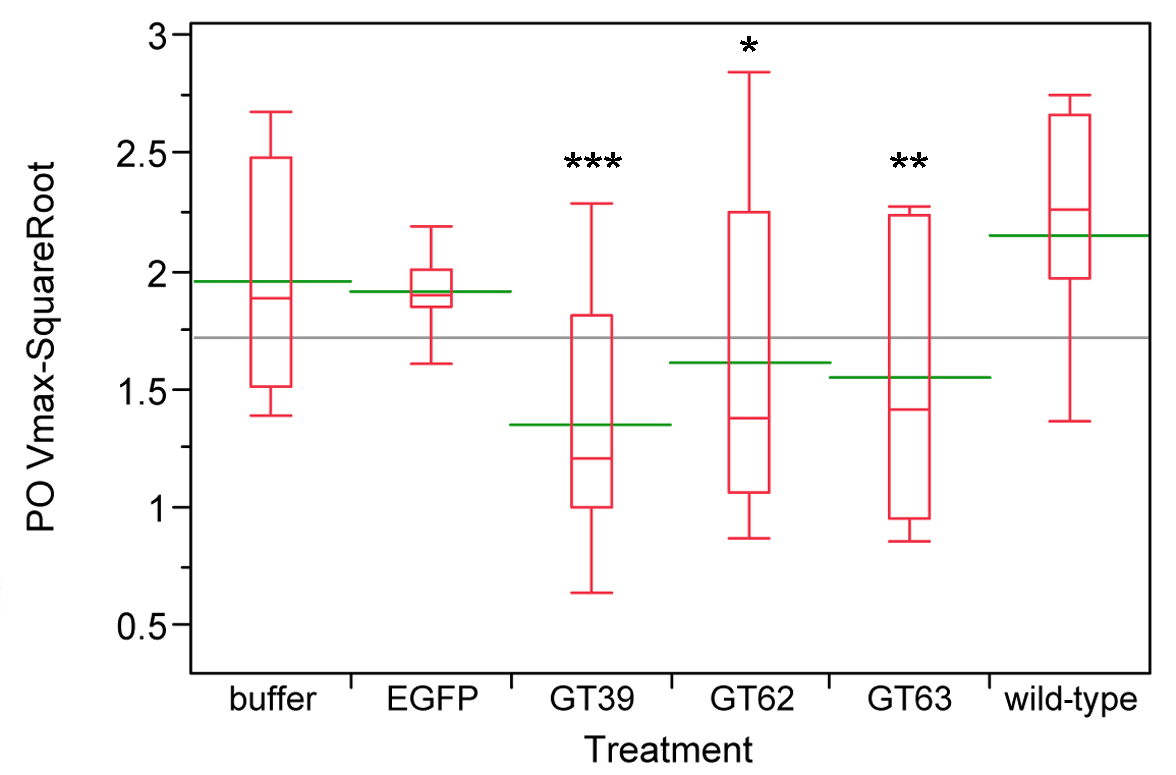

Supplement: Figure S3 — Phenol oxidase (PO) activity assays of wild-type and novel quinone-less gene RNAi knock-downs in males. The Y-axis indicates the square root of PO Vmax, red boxes are boxplots, green lines represent the mean value, the gray line represents the grand mean, while the X-axis presents wild-type, control injections, and different RNAi-knock-downs (N = 12–15, but the buffer-injected had only 4 beetles). Buffer: buffer-injection control; EGFP: dsEGFP-injection control; GT39: Tcas-ql VTGl; GT62: Tcas-ql ARSB; GT63: Tcas-ql MRP. The asterisks (*) marked the t-test results comparing to wild-type: ***, p<0.001; **, 0.001<p<0.01; *, 0.01<p<0.05. Buffer- and EGFP-injected controls were not significantly different from wild-type. (TIF) [file pgen.1003596.s013.tif]
