# Supplementary material for: Odoriferous Defensive Stink Gland Transcriptome to Identify Novel Genes Necessary for Quinone Synthesis in the Red Flour Beetle, Tribolium castaneum
Source: PLoS Genet. 2013 Jul 11;9(7):e1003596. doi: 10.1371/journal.pgen.1003596 (PMC3708791; doi:10.1371/journal.pgen.1003596)
Supplement: Text S1 — In a separate file, we provide descriptions on beetle culture, gland cytology, RNA extraction and cDNA library construction, cloning of 77 candidate genes, RACE PCR, photo imaging and processing, gas chromatography and mass spectrometry (GC-MS), microbe culturing for inhibition assays, gland whole mount fluorescent in situ hybridization (GWMFISH), and related references. (DOCX) [file pgen.1003596.s014.docx]

Text S1: Supplementary Materials and Methods

**Beetle culture**

Experiments were performed with the wild-type *Tribolium castaneum* strain San Bernardino. Animals were kept at 25°C, 40-60% relative humidity. After dsRNA injection, they were maintained at 32.5°C until phenotypic analysis.

**Gland cytology**

Odoriferous glands were dissected out of adult beetles (during this process the gland secretions in the reservoir are lost) and washed 3 times (5min each) with PBS in a 24-well cell culture plate, then incubated in DAPI solution (SIGMA-ALDRICH Chemie GmbH, Munich, Germany, Cat. No. D9542, final concentration 0.1µg/mL in PBS) for 20min. After incubation, glands were washed 3 times (10min each) with PBS again, and embedded in 80% glycerol for observation and photo imaging under Zeiss Axio Observer Z1 inverted fluorescent microscope (Carl Zeiss, Oberkochen, Germany).

**RNA extraction and cDNA library construction**

Adult total RNA was extracted by using TRIzol reagent (Invitrogen, Life Technologies GmbH, Darmstadt, Germany, Cat. No. 15596-018) following manufacturer’s instructions from a mixture of different adult stages (A0-A30), pre-adult and late pupal stages were also included in order to cover all the potential adult developmental genes. Then poly(A) was purified with the MicroPoly(A)Purist Kit (Ambion, Life Technologies GmbH, Darmstadt, Germany, Cat. No. AM1919). Gland total RNA was prepared as preparing the mRNA-seq materials from same stages. The concentrations were measured on NanoDrop spectrometer, and the qualities were checked on agarose gels. Double stranded cDNA libraries were constructed with SMART PCR cDNA Synthesis Kit (Clontech, Saint-Germain-en-Laye, France, Cat. No. 634902) according to the user manual.

**Cloning of 77 candidate genes**

The chosen 77 candidate genes are listed in ***Dataset S5***, as well as their primers and annealing temperatures for amplification using either Phusion High-Fidelity DNA Polymerase (Finnzymes, Thermo Fisher Scientific, Inc., Waltham, USA, Cat. No. F-530) or Advantage 2 PCR Enzyme System (Clontech, Saint-Germain-en-Laye, France, Cat. No.PT3281-1) from the adult cDNA library. Amplified fragments were ligated to PCR vectors with CloneJET PCR Cloning Kit (Fermentas, Thermo Fisher Scientific, Inc., Waltham, USA, Cat. No. K1231) or TA Cloning Kit Dual Promoter (pCRII) (Invitrogen, Life Technologies GmbH, Darmstadt, Germany, Cat. No. K2070).

**RACE PCR**

RACE cDNA template was prepared from adult poly(A) RNA with SMART RACE cDNA Amplification Kit (Clontech, Saint-Germain-en-Laye, France, Cat. No. 634914) according to the user manual. The specific primers were designed based on the amplified fragments and known sequences using Primer Premier 5.0 [1] according to the specifications in the manual of the kit and are listed in ***Dataset S5***.

**Photo imaging and processing**

During dissection after RNAi, the abnormal glands were recorded using a CCD camera linked with a stereomicroscope Leica MZ16FA (Leica Microsystems GmbH, Wetzlar, Germany). Then the photos were processed with Adobe Photoshop CS2.

**Gas chromatography and mass spectrometry**

The gas chromatography and mass spectrometry (GC-MS) system consisted of a 6980N gas chromatograph and a 5973N mass spectrometer from Agilent Technologies (Santa Clara, USA) together with an MPS autosampler from Gerstel (Mülheim, Germany). The samples were measured as soon as possible after preparations. During the analysis, the samples were kept in a cooled autosampler rack at ~10°C. One microliter of each sample was injected to the system. A capillary column HP-5ms (Agilent Technologies) was used (length 30 m, I.D. 0.25 mm, film thickness 0.25 µm). The split/splitless injector was operated at 250°C in the splitless mode. The carrier gas used was helium with a constant flow of 1.0 ml/min, which is equivalent to 36 cm/sec. The following temperature program was used: initial temperature 50°C, hold for 1.5 min, then with a rate of 7.5°C/min to 200°C, which was maintained for 5 min. Total run time was 26.5 min. The mass spectrometer was used in the scan mode (mass range 20–345 u). The data were analysed with the software MSD ChemStation D.02.00.275 (Agilent Technologies, Santa Clara, USA). Substance identification was performed with the NIST 2008 and Wiley 9^th^ edition databases (National Institute for Standards and Technology, Gaithersburg, USA / Wiley, Hoboken, USA). When available, the identification was verified with authentic standards.

**Microbe culturing for inhibition assays**

*Aspergillus niger* was maintained at 25°C on plates of Potato Extract Glucose Broth (Carl-Roth GmbH & Co. KG, Karlsruhe, Germany, Cat. No. CP74.1) with 15g/L Agar Bacteriological Oxoid No.1 (Oxoid, Thermo Fisher Scientific, Inc., Waltham, USA, Code LP0011). After sporulation of the fungi, spores were scraped off in Ringer’s solution (128mM NaCl, 18mM CaCl_2_, 1.3mM KCl, 2.3mM NaHCO_3_) using a pipette tip (3mL each plate). The spore suspension was diluted 10 times with Ringer’s and used for inoculation (1mL dilution for 10 mL medium). Reduced agar concentration was used for the assay plates (6g/L, 6mL per Ø9cm plate).

*Arthrobacter globiformis* was activated from lyophilization and cultured at 28°C overnight in CASO broth (Carl-Roth GmbH & Co. KG, Karlsruhe, Germany, Cat. No. X938.1). OD value was measured using UV spectrometer. According to the OD, culture was diluted to a final OD of 0.6. Then 1mL dilution was added to 250mL CASO (10g/L agar) to make assay plates (also 6mL per Ø9cm plate).

**Gland whole mount fluorescent *in situ* hybridization**

**Probe preparation.** Sense and anti-sense Digoxigenin (DIG) labeled probes were synthesized from gel extraction purified PCR products, which were amplified with T7 and T3-pJET-R primers, by using DIG RNA Labeling Mix (Roche, Cat. No. 11277073910), T3 RNA Polymerase (Roche Applied Science, Roche Diagnostics Deutschland GmbH, Mannheim, Germany, Cat. No. 11031163001) or T7 RNA Polymerase (Roche Applied Science, Roche Diagnostics Deutschland GmbH, Mannheim, Germany, Cat. No. 10881767001) as instructed by the user manuals. Then alkaline hydrolysis was used to hydrolyze the probes. Equal volume of carbonate buffer (120 mM Na_2_CO_3_, 80 mM NaHCO_3_, pH 10.2) was added to the probe and incubated at 60°C for 30 min. Six volumes of hybridization buffer [hyb-buffer: 50% formamide, 5 x SSC (pH 5.5), 100 µg/mL salmon sperm DNA, 100 µg/mL heparin, 0.1% Tween-20] were then added to halt the reaction [2]. Probes were stored at -20°C until use (-80 °C is suggested for long time storage).

**Gland whole mount fluorescent *in situ* hybridization (GWMFISH).** Glands were dissected in chilled phosphate-buffered saline [PBS: 145 mM NaCl, 1.4 mM KH_2_PO_4_, 8 mM Na_2_HPO_4_ (pH 7.4)] on ice and placed in 4% PFA (paraformaldehyde, in PBS) in a 24-well culture plate. When enough glands were collected, they were fixed for 30-40 min at RT or overnight at 4°C, washed twice in PBS, 15min each, then once in 50% PBS-methanol, twice in 100% methanol, and twice in ethanol for dehydration. Fixed glands were stored at -20°C for months or used right away. Glands were rehydrated through washing in 50% ethanol/PBT (PBS with 0.1% Tween-20) twice and then three times in PBT. A 6 min proteinase K (5 µg/mL in PBT) digest was followed by washes in PBT with 2 mg/mL glycine. After two washes in PBT, the tissues were post-fixed in 4% PFA for 40-60 min without agitation. The tissues were then washed three to four times in PBT and subsequently transferred to pre-warmed (66°C) W1 buffer [50% formamide, 5 x SSC (pH5.5), 1% SDS] for 5 min, then to the pre-warmed hyb-buffer. After at least 1 h of incubation in hyb-buffer at 66°C, the probe was mixed with hyb-buffer at a concentration of 2-10 ng/µl and heated to 95°C for 2 min, then placed on ice 5 min, and pre-warmed to 66°C.

After incubation with the probe for 14–48 h, the probe was removed, and the glands were washed three times 30 min each (rinse once before the first wash) in pre-warmed W1 buffer at 66°C, then overnight. The next day, the glands were washed twice with W2 Buffer (50% formamide, 2 x SSC, 1% SDS) at 66°C, once at RT, rinsed with W3 Buffer (2 x SSC, 0.1% Tween-20), and washed twice 10 min each. Optionally an RNAse treatment (0.02 mg/mL in W3 buffer, 37°C 20 min, then two times washes with W3) was performed. After an additional rinse in W4 Buffer (0.2 x SSC, 0.1% Tween-20), the tissues were washed in MABT buffer (100 mM maleic acid, 150 mM NaCl, 0.1% Tween-20, pH 7.5, fresh made) two times and blocked for 1 h at room temperature in blocking solution (2 mg/mL BSA and 10% sheep serum in MABT, fresh made). Glands were incubated overnight at 4 °C with anti-DIG-alkaline phosphatase (AP) Fab fragments (Roche Applied Science, Roche Diagnostics Deutschland GmbH, Mannheim, Germany, Cat. No. 11093274910) at a concentration of 1:3000. After washing with MABT buffer several times and then with detection buffer (100mM Tris-HCl, 100 mM NaCl, 10mM MgCl_2_, pH8.0), the color reaction was performed using HNPP Fluorescent Detection Set (Roche Applied Science, Roche Diagnostics Deutschland GmbH, Mannheim, Germany, Cat. No. 11758888001). Glands were rinsed in PBS to stop the color reaction and counterstained with Hoechst 33342 (SIGMA-ALDRICH Chemie GmbH, Munich, Germany, Cat. No. B2261) prior to mounting and embedding in Aqua-Poly/Mount (Polyscience, Niles, Illinois, USA, Cat. No. 18606). The stainings were observed and captured with a confocal laser scanning microscope Zeiss LSM780. 3D construction was performed using software ZEN2011 (Carl Zeiss MicroImaging GmbH, Oberkochen, Germany). Contrast and brightness were adjusted using Adobe Photoshop CS2. All washes were carried out with gentle agitations for 15min unless otherwise described.

Supplementary References

1. Lalitha S (2000) Primer Premier 5. Biotech Software & Internet Report 1: 270–272. Available:http://online.liebertpub.com/doi/abs/10.1089%2F152791600459894.

2. Osborne P, Dearden PK (2005) Non-radioactive in-situ hybridisation to honeybee embryos and ovaries. Apidologie 36: 113–118. doi:10.1051/apido.

3. Happ GM (1968) Quinone and hydrocarbon production in the defensive glands of Eleodes longicollis and Tribolium castaneum (Coleoptera, Tenebrionidae). Journal of insect physiology 14: 1821–1837. Available:http://www.sciencedirect.com/science/article/pii/002219106890214X.

4. Eisner T, McHenry F, Salpeter MM (1964) Defense mechanisms of arthropods. XV. Morphology of the quinone‐producing glands of a tenebrionid beetle (ELEODES longicollis lec.). Journal of morphology 115: 355–399. Available:http://onlinelibrary.wiley.com/doi/10.1002/jmor.1051150304/abstract. Accessed 4 June 2012.

5. Beeman RW, Stuart JJ, Haas MS, Friesen KS (1992) Chromosome extraction and revision of linkage group 2 in Tribolium castaneum. The Journal of heredity 87: 224–232. Available:http://www.ncbi.nlm.nih.gov/pubmed/8683098.

6. Suzuki T, Huynh V, Muto T (1975) Hydrocarbon repellents isolated from Tribolium castaneum and T. confusum (Coleoptera: Tenebrionidae). Agricultural and Biological Chemistry 39: 2207 – 2211. Available:http://agris.fao.org/agris-search/search/display.do?f=1976/JP/JP76004.xml;JP7601784.

7. Görgen G, Frößl C, Boland W, Dettner K (1990) Biosynthesis of 1-alkenes in the defensive secretions of Tribolium confusum (Tenebrionidae); stereochemical implications. Experientia 46: 700–704. Available:http://www.springerlink.com/content/hx288200k15331hr/. Accessed 10 August 2012.
